# Hints on T cell responses in a fish-parasite model: *Enteromyxum leei* induces differential expression of T cell signature molecules depending on the organ and the infection status

**DOI:** 10.1186/s13071-018-3007-1

**Published:** 2018-07-31

**Authors:** M. Carla Piazzon, Itziar Estensoro, Josep A. Calduch-Giner, Raquel del Pozo, Amparo Picard-Sánchez, Jaume Pérez-Sánchez, Ariadna Sitjà-Bobadilla

**Affiliations:** 1 Fish Pathology Group, Institute of Aquaculture Torre de la Sal (IATS-CSIC), Ribera de Cabanes, Castellón, Spain; 2 Nutrigenomics and Fish Growth Endocrinology Group, Institute of Aquaculture Torre de la Sal (IATS-CSIC), Ribera de Cabanes, Castellón, Spain

**Keywords:** Myxozoa, Teleost, Gilthead sea bream, T lymphocytes, Cytokines

## Abstract

**Backgroud:**

*Enteromyxum leei* is a myxozoan parasite that produces a slow-progressing intestinal disease. This parasite invades the paracellular space of the intestinal epithelium and progresses from the posterior to the anterior intestine. The aim of the present study was to gain insights into fish T cell responses in the gilthead sea bream-*E. leei* infection model using a PCR-array with 30 signature molecules for different leukocyte responses in head kidney, spleen, anterior and posterior intestine.

**Results:**

The PCR-array results suggest that *E. leei* induced migration of T cells from head kidney to intestines where T_H1_, CTL and T_H17_ profiles were activated and kept in balance by the upregulation of regulatory cytokines. These results were partially validated by the use of cross-reacting antibodies and BrdU immunostaining to monitor proliferation. Zap70 immunostaining supported the increased number of T cells in the anterior intestine detected by gene expression, but double staining with BrdU did not show active proliferation of this cell type at a local level, supporting the migration from lymphohaematopoietic tissues to the site of infection. Global analyses of the expression profiles revealed a clear separation between infected and exposed, but non-infected fish, more evident in the target organ. Exposed, non-infected animals showed an intermediate phenotype closer to the control fish.

**Conclusions:**

These results evidence a clear modulation of the T cell response of gilthead sea bream upon *E. leei* infection. The effects occurred both at local and systemic levels, but the response was stronger and more specific at the site of infection, the intestine. Altogether, this research poses a promising basis to understand the response against this important parasite and establish effective preventive or palliative measures.

**Electronic supplementary material:**

The online version of this article (10.1186/s13071-018-3007-1) contains supplementary material, which is available to authorized users.

## Background

Gilthead sea bream (*Sparus aurata*), a marine teleost species, is the main farmed fish in the Mediterranean [1]. One of the pathogens that threatens gilthead sea bream culture is the myxozoan intestinal parasite *Enteromyxum leei*. Myxozoans are obligate endoparasitic metazoans belonging to the phylum Cnidaria. Currently, more than 2000 myxozoan species have been described. They typically present a two-host life-cycle involving invertebrates and vertebrates as definitive and intermediate hosts, respectively [2]. The invertebrate host of *E. leei* is still unknown, but fish-to-fish transmission is feasible [3]. *Enteromyxum leei* slowly and progressively invades the intestinal epithelium of the host inducing loss of appetite and poor food conversion rates, leading to macroscopic disease signs such as emaciation, diminished growth and condition factor, cachexia and eventually death [4]. The parasite colonizes first the posterior intestinal segment and progresses to the anterior portion invading the middle intestine lastly [4]. Currently, there are no preventive or curative measures against this disease. Thus, several studies have been conducted to understand the immune responses elicited by the parasite in order to manage infections. *E. leei* induces a massive hyperplasia of the intestinal lamina propria-submucosa due to recruitment and proliferation of heterogeneous leukocytes [5]. More specifically, *E. leei* is known to induce B cell responses at a local level, with increased numbers of intestinal IgM^+^ B cells and increased transcription of secreted and membrane *IgM* and *IgT* [6, 7]. Recruitment of mast cells and depletion of acidophilic granulocytes have also been described in infected gilthead sea bream intestine [8]. Interleukin gene expression profiles elicited by *E. leei* infections were characterized by an early pro-inflammatory profile that later switched to an anti-inflammatory pattern in infected posterior intestinal segments [9]. Indisputably, this parasite regulates the immune response, mainly at a local level (intestine), but also systemically. The progression pattern of the disease, where the parasite is only present at the anterior intestine at later infection stages, indicates that different responses are taking place at the different intestinal segments. So far, the T cell response in this infection model has not been characterized. Thus, this study constitutes the first step for understanding the T cell response of gilthead sea bream upon infection with *E. leei*.

T lymphocytes are critical components of the adaptive immune system in vertebrates. They recognize specific antigens through the T cell receptor (TCR). The TCR is associated with the CD3 complex, responsible for the intracellular signal transduction, which includes recruitment and activation of ZAP70 and LCK kinases [10]. T cells are subdivided in two major subtypes depending on their function. CD8^+^ cytotoxic T lymphocytes (CTLs) directly kill cells by the crosslinking of death receptors such as Fas [11] or by releasing cytolytic effector molecules such as perforin or granzymes [12]. CD4^+^ helper T cells (T_H_) coordinate specific immune responses by releasing different types of cytokines depending on the nature of the invading microorganism or threat. In mammals, upon activation, CD4^+^ T cells proliferate and differentiate into effector T_H_ cells. T_H_ cells can be further subdivided in different subsets, including T_H1_, T_H2_, T_H17_ and T_reg_, according to their distinct cytokine secretion patterns and their immunomodulatory effect [13]. These subsets represent different states of activation with certain degrees of plasticity rather than an endpoint terminal differentiation [14, 15]. T_H1_ fate is driven by the transcription factor Tbet and is aimed to control intracellular infections by producing effector cytokines such as IFNγ and TNFα and inducing CTLs. T_H2_ cells are characterized by the expression of the transcription factor GATA3 and the release of cytokines like IL4 and IL13, mediating B cell activation and antibody production to face extracellular infections [16]. T_H17_ cells express the transcription factor RORγ and secrete IL17A, IL17F, IL21 and IL22 to control extracellular bacteria and fungi [17]. FOXP3 is the transcription factor expressed in T_reg_ cells. T_reg_ cells are implicated in the regulation of the immune response and maintaining self-tolerance by producing regulatory cytokines such as IL10 and TGFβ [18]. It is important to mention that the different subsets are not exclusive. The presence or regulation of a certain cytokine does not indicate the absolute occurrence of a particular T cell subtype. T cell responses are complex and the expression of multiple cytokines, surface molecules and transcription factors are needed to draw a full picture of the type of response that can be occurring.

Homologs of most of the surface markers, cytokines and transcription factors associated with T cell responses have been identified in many teleost species [19, 20]. To date, we cannot assert that fish and mammalian T cell responses are equivalent, but the functional studies conducted so far indicate a strong degree of conservation of these phenotypes [21].

The aim of the present study was to gain insight into fish T cell responses by using the gilthead sea bream-*E. leei* infection model and the expression pattern of an extensive newly designed panel of signature genes for different T cell responses. Markers for B cells and other leukocytes were also studied. The parallel use of cross-reacting commercial antibodies allowed for the validation of the expression results for some markers (Zap70 and Tbet) at protein levels. The overall picture obtained from this study improves our currently limited knowledge on fish T cells and defines how this response can be regulated in the intestine upon a parasitic infection.

## Methods

### Fish, experimental infection and sampling procedure

Gilthead sea bream juvenile specimens (mean weight ± SEM 13.7 ± 0.27 g) from a commercial fish farm were checked by PCR (*18S* ribosomal RNA gene) and histological analyses [4, 22] to be specific pathogen free and clinically healthy, and were transported to the IATS-CSIC facilities (Castellón, Spain). Fish were kept in 5 μm-filtered sea water, with natural photoperiod and temperature (ranging from 22 to 26.5 °C) and fed *ad libitum* with a commercial diet throughout all the experiment. After a 6-week acclimatization period, 100 fish with an average weight of 24.4 g (SEM = 0.99 g), were allocated in four 90 l tanks (25 fish/tank). All tanks had the same conditions of temperature, water quality and oxygen concentration. Day-length and water temperature followed the natural changes at IATS latitude (40°5'N, 0°10'E, ranging from 22 to 26.5 °C over the course of the experiment) and the salinity of seawater was 37.5 g/l. The oxygen content of outlet water always remained higher than 75% saturation. Fish were starved for 48 h and animals from two replicated tanks were intubated anally with 0.2 ml of *E. leei*-infected intestinal scrapings [recipient (RCPT) groups] as described in [3]. Fish in the other two replicated tanks were intubated with the same volume of PBS to constitute the control (CTRL) groups. Ten weeks post-intubation (p.i.), 5 fish/tank (10 fish/group) were sacrificed with an overdose of the anaesthetic MS-222 (0.1 g/l; Sigma-Aldrich, St. Louis, MO, USA)) and pieces of head kidney, spleen, anterior and posterior intestines were collected in RNAlater (Qiagen, Hilden, Germany) and stored at 4 °C until RNA isolation. In parallel, samples of head kidney, spleen, anterior, middle and posterior intestine were fixed in 10% buffered formalin for histological procedures. Samples from each fish were individually identified to compare results obtained from the different assays.

### RNA extraction and reverse transcription

RNA from head kidney, spleen, anterior and posterior intestines was extracted using MagMAX™-96 total RNA isolation kit (Applied Biosystems, Foster City, CA, USA ). The RNA concentration and quality was determined using a Nanodrop 2000c (Thermo Scientific, Wilmington, DE, USA) and the integrity was assessed on an Agilent 2100 Bioanalyzer (Santa Clara, CA, USA). RNA integrity number (RIN) values were always between 8 and 10. To avoid genomic DNA contamination, 500 ng of RNA from each sample were treated with DNaseI amplification grade (Invitrogen, Carlsbad, CA, USA ) prior to reverse transcription. Reverse transcription of 500 ng of input RNA was performed using the High-Capacity cDNA Archive Kit (Applied Biosystems). All procedures were performed following each manufacturer’s instructions.

### Gene expression analyses

Real-time (RT) quantitative PCR was carried out using the CFX96 Connect™ Real-Time PCR Detection System (Bio-Rad, Hercules, CA, USA) in a 96-well layout designed for simultaneously profiling the genes of interest for each tissue and individual. The primers used in this study (Additional file 1: Table S1) were selected to detect target genes characteristic of the specific responses to be studied. The primers were designed and/or checked for specificity by datamining of the gilthead sea bream transcriptomic database [23] (http://nutrigroup-iats.org/seabreamdb/). Out of the 30 sequences used in this study, 16 were described for the first time in gilthead sea bream and uploaded to GenBank (Additional file 1: Table S1). All primers were checked to have similar efficiencies and higher than 90% (ranging from 90.17 to 102.69), and the correct product size was assessed in a 1% agarose gel. Each RT reaction of 20 μl contained 3.3 ng of total input cDNA sample, 5× PyroTaq EvaGreen qPCR Mix Plus (Cultek Molecular Bioline, Madrid, Spain) and specific primers at a final concentration of 0.45 μM. The PCR reaction conditions consisted of an initial denaturation step at 95 °C for 3 min, followed by 40 cycles of denaturation for 15 s at 95 °C and annealing/extension for 60 s at 60 °C. The specificity of the reactions was verified by visual analysis of melting curves for each reaction performed. Fluorescence data acquired during the PCR extension phase were normalized by the delta-delta Ct method [24]. Four potential housekeeping genes (*β-actin*, *elongation factor 1α*, *α-tubulin* and *18S rRNA*) were previously tested for stability using the GeNorm software. The most stable reference gene among conditions in each tissue was *β-actin* and it was used in the normalization procedure.

### Histological infection diagnosis

Parasite diagnosis was performed on anterior, middle and posterior intestinal segments fixed in 10% buffered formalin, embedded in paraffin, 4 μm-sectioned and stained with Giemsa following standard procedures. Infection intensity was semiquantitatively evaluated in each intestinal segment using a scale from 1 (lowest) to 6 (highest) as previously described [3]. Non-infected segments were scored as 0.

### Immunohistochemical analyses

To validate the PCR-array results, the sequences of the gilthead sea bream T cell specific molecules were checked for homology with their mammalian counterparts in order to identify commercial cross-reacting antibodies to be used in immunohistochemical studies. This search produced two candidate antibodies, one against Zap70 [Zap-70 (99F2) Rabbit mAb; Cell Signaling Technologies, Leiden, The Netherlands] that had already been validated for other fish species [25], and other against Tbet (TBX21 PA5-28881 Rabbit pAb; Thermo Fisher, Rockford, IL, USA). The epitopes were more than 75% similar with long stretches of identical amino acids between gilthead sea bream and the target species. Possible cross-reactivity with undesired proteins was ruled out by BLAST analysis using the gilthead sea bream transcriptomic database [23] (http://nutrigroup-iats.org/seabreamdb/). In all immunohistochemical studies, a negative control without the primary antibody was included.

In order to characterize actively proliferating cells, bromodeoxyuridine (BrdU) DNA labelling was performed as previously described [26]. Briefly, 24 h prior to sampling, fish were intracoelomically injected with 100 mg 5-bromo-2’-deoxyuridine (BrdU; Sigma-Aldrich, St. Louis, MO, USA per kg of fish weight).

Samples of anterior and posterior intestine from three animals that showed high (RCPT4, RCPT5 and RCPT7) and low (CTRL4, CTRL5 and CTRL10) expression levels of *zap70* and *tbet* (see Additional file 2: Table S2) were selected for immune staining. Four-micrometer-thick paraffin sections were collected on Super-Frost-plus microscope slides (Menzel-Gläser, Braunschweig, Germany), dried overnight, deparaffinised and hydrated. All incubations were performed in a humid chamber, at room temperature. The washing steps consisted of 5 min immersion in TTBS (20 mM Tris-HCl, 0.5 M NaCl, 0.05% Tween 20, pH 7.4) and 5 min immersion in TBS (without Tween 20), unless otherwise stated. An antigen retrieval step was performed by boiling the samples in citrate buffer pH 6 for 20 min followed by one washing step in TBS. The endogenous peroxidase activity was blocked by incubation in hydrogen peroxide 0.3% v/v in distilled water (H_2_O:H_2_O_2_ in a 9:1 proportion) for 30 min. Slides were washed and blocked 30 min with TBS 1.5% normal goat serum (Vector Laboratories, Burlingame, CA, USA). After washing, they were incubated with the rabbit anti-Zap70 or rabbit anti-Tbet antibodies 1:50 and 1:100, respectively, in TBS 1% BSA for 2 h. Then, samples were washed again and incubated with a biotinilated goat anti-rabbit antibody (Vector Labs) 1:200 in TBS 1.5% normal goat serum for 1 h. The slides were subsequently washed, incubated for 1 h with the avidin-biotin-peroxidase complex (ABC, Vector Labs), washed and developed by incubating with 3,3’-diaminobenzidine tetrahydrochloride chromogen (DAB, Sigma-Aldrich, St. Louis, MO, USA) for 2–4 min. The reaction was stopped with deionized water and the slides were counterstained for 5 min with Gill’s haematoxylin before being dehydrated and mounted for light microscopy examination.

To assess whether the higher number of Zap70^+^ cells observed in infected anterior intestine were due to active proliferation of resident cells, a double staining with anti-BrdU and anti-Zap70 antibodies was performed as previously described [26] with some modifications. After antigen retrieval, slides were incubated in HCl 2 N for 30 min, blocked in PBS, 5% BSA, 0.5% Triton X-100 and subsequently incubated for two hours with mouse Mab anti-BrdU clone BU-33 (1:500, Sigma) and rabbit anti-Zap70 (1:50) in PBS at room temperature (RT). After two washes for 5 min with PBS 0.1% Tween 20, the samples were incubated for 1 h with 10 μg/ml of goat anti-mouse alexa fluor 488 (Invitrogen) and goat anti-rabbit Texas red (Vector Labs), washed again, stained with DAPI and mounted in aqueous mounting medium for fluorescence microscopy examination. For this study, the anterior intestines of three fish with high expression of *zap70* and high number of Zap70^+^ cells (RCPT4, RCPT5 and RCPT7) were used. Three samples per fish were examined in order to determine the number of double positive cells.

### Data processing and statistical analyses

Differences in gene expression were calculated comparing RCPT fish (*n* = 10) with CTRL fish (*n* = 10) values using GraphPad PRISM v.5.03 and R statistical software v.3.0.2 [27]. For statistical analyses, gene expression data were log-transformed (LN). For normally distributed data, differences were evaluated using Student’s t-test and one-way ANOVA followed by the Tukey *post-hoc* test for multiple comparisons. When conditions were not met, non-parametric tests (Mann-Whitney-Wilcoxon or Kruskal-Wallis followed by Dunn’s test) were used. The significance level was set at *P* < 0.05 unless otherwise stated. Principal components analyses (PCA) were performed using individual gene expression values and the default *prcomp* R function. Hierarchical cluster analyses were performed using the default *hclust* R function. Visualizations were constructed using the *factoextra* (v.1.0.4) and *gplots* (v.3.0.1) R packages. Heatmaps were made using mean values for each gene, tissue and group using the *heatmap.2* function from the *gplots* R package centring and scaling the values per row.

## Results

### Infection status

Ten weeks after *E. leei* anal intubation, the prevalence of infection in the 10 sampled fish/group was 70% in the RCPT group and 0% in the CTRL group. The RCPT group showed significantly lower weight, size and condition factor than the CTRL group, evidencing the typical infection signs induced by this parasite [4] (Table 1). The seven RCPT fish that were *E. leei*-positive by histological observations (RCPT^+^) showed even more pronounced disease signs, particularly weight and size (Table 1) and high intensity of infection in the posterior intestine. Two of these fish were also positive in the anterior intestine, and only one scored positive in the middle intestine (Table 2). RCPT fish negative for the parasite (RCPT^−^) showed less pronounced disease signs with size (ANOVA: *F*_(2, 17)_ = 22.67, *P* < 0.0001) and weight (ANOVA: *F*_(2, 17)_ = 21.65, *P* < 0.0001) significantly higher than the RCPT^+^ group and not significantly different from the CTRL group, and an intermediate condition factor between RCPT^+^ and CTRL fish (Table 1).

**Table 1:** Recipient (RCPT) fish showed characteristic disease signs of *Enteromyxum leei* infection. Mean prevalence of infection, weight, size and condition factor (100 × weight/size^3^) of the fish used in this study

Group	Prevalence of infection (%)	Mean weight± SEM (g)	Mean size ± SEM (cm)	Mean condition factor ± SEM
CTRL	0	85.41 ± 6.54^a^	14.55 ± 0.37^a^	2.71 ± 0.04^a^
RCPT	70	64.95 ± 6.89*	13.37 ± 0.43*	2.61 ± 0.06*
RCPT^+^	100	59.43 ± 7.70^b^	13.04 ± 0.52^b^	2.58 ± 0.07^b^
RCPT^−^	0	77.83 ± 13.41^a^	14.17 ± 0.60^a^	2.67 ± 0.15^a,b^

Gilthead sea bream were exposed (RCPT, *n* = 10) or not (CTRL, *n* = 10) to the parasite *E. leei* by anal intubation and sampled after 10 weeks. Asterisks (*) indicate significant differences with the CTRL group (t-test, *P* < 0.05). The analysis was also performed separating RCPT fish positive (RCPT^+^, *n* = 7) and negative (RCPT^−^, *n* = 3) for the parasite by histological diagnosis (Table 2). Different superscript letters indicate statistical differences among groups for each of the parameters (ANOVA, *P* < 0.05)

**Table 2:** Evaluation of infection intensity per intestinal segment in recipient (RCPT) fish. *Enteromyxum leei* infection was semiquantitatively evaluated using a scale from 0 (no parasite observed in the segment) to 6 (maximum infection intensity). All CTRL fish scored 0 in all segments

Fish	AI	MI	PI
RCPT1	6	6	6
RCPT2	0	0	6
RCPT3	0	0	6
RCPT4	0	0	0
RCPT5	0	0	6
RCPT6	0	0	0
RCPT7	0	0	5
RCPT8	0	0	6
RCPT9	0	0	0
RCPT10	3	0	5

*Abbreviations*: AI, anterior intestine segment; MI middle intestine segment; PI, posterior intestine segment

### Leukocyte markers are differentially expressed upon *Enteromyxum leei* infection

The expression of 21 T cell master transcription factors, receptors, enzymes and signature cytokines was used to identify the T cell responses in gilthead sea bream head kidney, spleen, anterior and posterior intestines upon infection with *E. leei*. Middle intestine was not included in the study because only one fish scored positive in this segment, thus no statistical analyses could be performed with the subsequent results. To complete the study, six B cell specific molecules and specific markers for nonspecific cytotoxic cells (NCCs), eosinophilic granulocytes and macrophages were included in the array (Additional file 1: Table S1). The individual expression relative to the *β-actin* for each of these 30 genes is shown in Additional file 2: Table S2.

*Enteromyxum leei* infection induced downregulation of T cell markers in head kidney. Both *pan* T cell markers [*zap70* (t-test: *t*_(18)_ = 6.497, *P* < 0.0001) and *cd3ζ* (t-test: *t*_(18)_ = 5.841, *P* < 0.0001)] and the specific T_H_ [*cd4-1* (t-test: *t*_(18)_ = 4.983, *P* < 0.0001) and *cd4-2* (t-test: *t*_(18)_ = 2.325, *P* = 0.03)] and CTL [*cd8α* (t-test: *t*_(18)_ = 3.427, *P* = 0.003) and *cd8β* (t-test: *t*_(18)_ = 3.694, *P* = 0.0017)] receptors were significantly downregulated in the head kidney of the RCPT group when compared to the CTRL group. No significant effect on T cell responses was found in spleen, but the significantly higher expression of *nccrp1* (t-test: *t*_(18)_ = 3.265, *P* = 0.0043), *epx* (t-test: *t*_(18)_ = 3.99, *P* = 0.0009) and *mpeg1* (t-test: *t*_(17)_ = 1.78, *P* = 0.09) points to a higher presence of NCCs, eosinophils and macrophages in RCPT spleen. Anterior intestine of RCPT fish showed a significant increase in T cell markers, for both T_H_ and CTLs cells (*zap70* t-test: t_(18)_ = 3.398, *P* = 0.0032; *cd3ζ* t-test: *t*_(15)_ = 4.461, *P* = 0.0005; *cd4-1* t-test: *t*_(17)_ = 2.729, *P* = 0.014; *cd4-2* t-test: *t*_(14)_ = 3.897, *P* = 0.0016; *cd8α* t-test: *t*_(18)_ = 2.631, *P* = 0.017; *cd8β* t-test: *t*_(18)_ = 2.602, *P* = 0.018). Type 1 signature transcription factors and cytokines (*tbet* t-test: *t*_(18)_ = 4.076, *P* = 0.0007, *tnfα* t-test: *t*_(18)_ = 1.837, *P* = 0.09 and *ifnγ* t-test: *t*_(18)_ = 5.55, *P* < 0.0001), *il17a*/*f* (t-test: *t*_(18)_ = 2.197, *P* = 0.041) and CTL receptors and enzymes (*cd8α*, *cd8β*, *gzma* (t-test: *t*_(18)_ = 4.869, *P* < 0.0001) and *prf1* (t-test: *t*_(18)_ = 1.741, *P* = 0.049)) were upregulated in RCPT anterior intestine, together with a general upregulation of B cell molecules (t-test: *mIgM* t-test: *t*_(18)_ = 2.538, *P* = 0.021; *IgD* t-test: *t*_(18)_ = 2.249, *P* = 0.037; *pax5* t-test: *t*_(17)_ = 3.017, *P* = 0.007; *sIgM* t-test: *t*_(18)_ = 2.364, *P* = 0.0295) and no change in innate cell markers. In the posterior intestine of RCPT fish, type 1 response signature genes (*tbet* t-test: *t*_(18)_ = 2.856, *P* = 0.0105; *ifnγ* t-test: *t*_(18)_ = 4.511, *P* = 0.0003), *il6* (t-test: *t*_(18)_ = 2.489, *P* = 0.0228), *il4*/*13a* (t-test: *t*_(18)_ = 2.26, *P* = 0.0365), *il17a*/*f* (t-test: *t*_(18)_ = 3.813, *P* = 0.0013) and the B cell markers were upregulated (*mIgM* t-test: *t*_(18)_ = 4.315, *P* = 0.0004; *pax5* t-test: *t*_(18)_ = 3.833, *P* = 0.0012; *sIgM* t-test: *t*_(18)_ = 4.389, *P* = 0.0004). The upregulation of *gzma* (t-test: *t*_(18)_ = 4.151, *P* = 0.0006), however, was not consistent with the downregulation of the other CTL markers (*gzmb* t-test: *t*_(18)_ = 2.411, *P* = 0.027; *prf1* t-test: *t*_(18)_ = 2.843, *P* = 0.011) (Table 3). Significant upregulation of *il10* was found in spleen (t-test: *t*_(18)_ = 2.345, *P* = 0.031), anterior (t-test: *t*_(18)_ = 3.49, *P* = 0.0026) and posterior (t-test: *t*_(17)_ = 2.541, *P* = 0.021) intestinal segments of RCPT animals.

**Table 3:** *Enteromyxum leei* exposure induces the most significant changes in intestine

Cell types	Gene	Head kidney	Spleen	Anterior intestine	Posterior intestine
T cells					
*Pan* T cell	*zap70*	0.63 ± 0.04*	0.82 ± 0.07	1.57 ± 0.15*	1.23 ± 0.13
	*cd3ζ*	0.62 ± 0.06*	1.09 ± 0.06	1.63 ± 0.16*	0.97 ± 0.09
T_H_ cells	*cd4-1*	0.50 ± 0.04*	0.91 ± 0.13	1.44 ± 0.16*	1.07 ± 0.11
	*cd4-2*	0.70 ± 0.11*	1.25 ± 0.18	1.59 ± 0.10*	1.22 ± 0.12
CTLs	*cd8α*	0.53 ± 0.06*	0.84 ± 0.10	1.92 ± 0.34*	0.89 ± 0.14
	*cd8β*	0.47 ± 0.07*	0.80 ± 0.16	1.69 ± 0.26*	0.79 ± 0.09
Transcription factors	*tbet*	1.03 ± 0.12	0.94 ± 0.09	2.17 ± 0.27*	1.67 ± 0.21*
	*gata3*	0.72 ± 0.07	0.82 ± 0.09	0.74 ± 0.11	1.22 ± 0.13
	*foxp3*	0.59 ± 0.06*	1.02 ± 0.14	0.99 ± 0.13	1.23 ± 0.20
Driver/effector cytokines	*tnfα*	1.04 ± 0.21	1.86 ± 0.43^0.08^	1.34 ± 0.13^0.09^	4.38 ± 2.96
	*ifnγ*	0.93 ± 0.08	1.05 ± 0.04	3.57 ± 0.45*	6.22 ± 1.15*
	*il12(p40)*	0.49 ± 0.06	1.53 ± 0.40	0.70 ± 0.12	0.62 ± 0.16^0.07^
	*il4/13a*	0.93 ± 0.19	1.89 ± 0.43^0.08^	1.07 ± 0.16	3.22 ± 0.97*
	*Il4/13b*	0.91 ± 0.07	0.72 ± 0.09^0.06^	0.91 ± 0.08	1.00 ± 0.09
	*il6*	2.09 ± 0.83	0.81 ± 0.13	1.95 ± 0.70	10.34 ± 3.75*
	*il10*	0.92 ± 0.09	1.34 ± 0.13*	2.04 ± 0.28*	1.56 ± 0.25*
	*tgfβ*	1.05 ± 0.15	1.27 ± 0.18	0.77 ± 0.09	0.54 ± 0.04*
	*il17a/f*	2.15 ± 1.27	2.08 ± 0.60	7.09 ± 2.75*	7.28 ± 1.63*
Cytotoxic enzymes	*gzma*	0.91 ± 0.19	1.73 ± 0.26^0.06^	9.32 ± 1.69*	10.97 ± 2.40*
	*gzmb*	1.13 ± 0.20	1.22 ± 0.15	1.10 ± 0.22	0.50 ± 0.07*
	*prf1*	0.89 ± 0.12	1.02 ± 0.07	2.08 ± 0.60*	0.54 ± 0.07*
B cells					
B cells	*mIgM*	0.81 ± 0.12	1.05 ± 0.16	2.92 ± 0.75*	3.55 ± 0.57*
	*mIgT*	0.68 ± 0.09^0.06^	0.58 ± 0.12*	1.65 ± 0.37	1.75 ± 0.47
	*IgD*	0.97 ± 0.12	0.90 ± 0.18	1.86 ± 0.36*	1.92 ± 0.50^0.09^
	*pax5*	0.93 ± 0.10	0.92 ± 0.13	1.59 ± 0.22*	2.49 ± 0.35*
Plasma cells	*sIgM*	1.05 ± 0.16	1.23 ± 0.15	13.54 ± 5.30*	7.46 ± 1.46*
	*sIgT*	0.44 ± 0.23	1.00 ± 0.22	10.35 ± 7.8^0.09^	104.29 ± 89^0.09^
NCC cells					
NCC	*nccrp1*	1.09 ± 0.11	1.93 ± 0.28*	0.87 ± 0.16	0.99 ± 0.11
Myeloid cells					
Eosinophils	*epx*	1.09 ± 0.08	3.62 ± 0.63*	2.44 ± 1.02	1.18 ± 0.31
Macrophages	*mpeg1*	1.15 ± 0.18	1.45 ± 0.27^0.09^	0.83 ± 0.11	0.92 ± 0.11

Mean ± SEM of the fold changes of the 30 studied genes in RCPT fish (*n* = 10) calculated relative to the CTRL group (*n* = 10) in each tissue. Bold values indicate significant up- (values > 1) or downregulation (values < 1). Asterisks (*) indicate significant differences at *P* < 0.05. Superscript numbers indicate the *P*-values between 0.05 and 0.1

To determine the T_H1_ / T_H2_ balance, the ratios *tbet* / *gata3* and *ifnγ* / *il4*/*13a* were calculated for each organ in CTRL and RCPT fish (Table 4). In CTRL fish, the profile of head kidney and spleen was T_H1_-skewed in comparison to intestine, which was T_H2_-skewed, as both ratios were significantly lower in intestine than in head kidney and spleen (*tbet* / *gata3* ANOVA: *F*_(3, 36)_ = 25.82, *P* < 0.0001; *ifnγ* / *il4*/*13a* ANOVA: *F*_(3, 36)_ = 7.572, *P* = 0.0005). When comparing CTRL with RCPT fish, the ratios did not change in lymphohaematopoietic tissues. In anterior intestine, a significant increase in both ratios (*tbet* / *gata3* t-test: *t*_(18)_ = 5.928, *P* < 0.0001; *ifnγ* / *il4*/*13a* t-test: *t*_(18)_ = 2.552, *P* = 0.02) indicated a shift to a T_H1_ profile. In the posterior intestine of RCPT fish, the *ifnγ* / *il4*/*13a* ratio increased (t-test: *t*_(18)_ = 2.883, *P =* 0.0099), but the *tbet* / *gata3* remained unchanged.

**Table 4:** *Enteromyxum leei* induces a shift towards a T_H1_ environment in the intestine of gilthead sea bream

Organ	Head kidney		Spleen		Anterior intestine		Posterior intestine	
Group	CTRL	RCPT	CTRL	RCPT	CTRL	RCPT	CTRL	RCPT
*tbet* / *gata3* ratio	3.90 ± 0.43^a^	5.10 ± 0.73	2.54 ± 0.40^a^	2.65 ± 0.23	0.53 ± 0.09^b^	1.45 ± 0.13*	0.94 ± 0.14^b^	1.13 ± 0.10
*ifnγ* / *il4* / *13a* ratio	6.72 ± 3.08^a^	4.16 ± 1.62	18.35 ± 5.14^a^	9.79 ± 2.45	1.06 ± 0.33^b^	2.63 ± 0.52*	0.59 ± 0.10 ^b^	1.74 ± 0.38*

The T_H1_ / T_H2_ balance was assessed as the ratio of *tbet* / *gata3* and *ifnγ / il4 / 13a* expression values for each individual fish in the study. Data are shown as mean ± SEM for *n* = 10 fish. Asterisks (*) indicate significant differences between *E. leei*-exposed (RCPT) and control (CTRL) groups within each organ (t-test, *P* < 0.05). Different superscript letters indicate significant differences in the CTRL groups among all organs within each calculated ratio (ANOVA, *P* < 0.05)

### Higher numbers of Zap70^+^ and Tbet^+^ cells are present in infected intestines

**Fig. 1:**
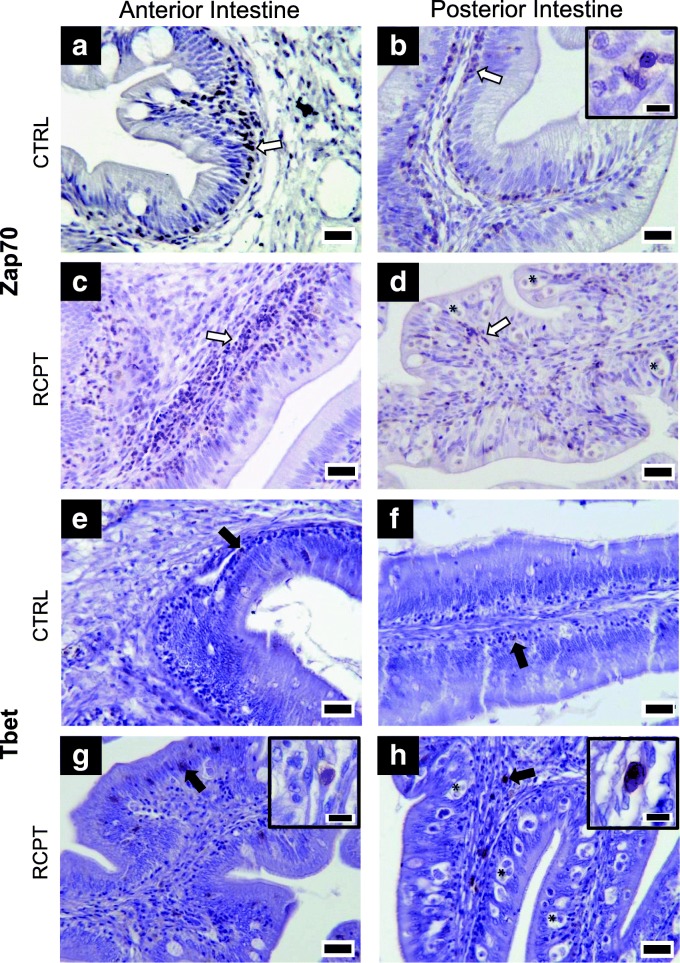
Zap70^+^ and Tbet^+^ cells are more abundant in *E. leei* infected fish. Representative micrographs of anterior (**a**, **c**, **e**, **g**) and posterior (**b**, **d**, **f**, **h**) intestines that showed either low [control fish (CTRL): **a**, **b**, **e**, **f**] or high [recipient fish (RCPT): **c**, **d**, **g**, **h**] *zap70* or *tbet* gene expression, stained with an anti-Zap70 (**a**, **b**, **c**, **d**) or an anti-Tbet (**e**, **f**, **g**, **h**) antibodies. White arrows point to some representative Zap70^+^ cells. Black arrows point to representative Tbet^+^ cells. Some parasites are labeled with asterisks (*) in the posterior intestine images (**d**, **h**). *Scale-bars*: 20 μm. Insets in **b**, **g** and **h** show immunoreactive cells at higher magnification. *Inset scale-bars*: 5 μm

The higher expression of *zap70* and *tbet* in infected fish intestine was also observed at protein levels by immunohistochemistry. A moderate number of Zap70^+^ lymphocyte-like cells were observed in CTRL anterior (Fig. 1a) and posterior (Fig. 1b) intestine. These cells were located in the base of the epithelium forming a one-cell-thick layer. Zap70^+^ cells were visibly more abundant in RCPT anterior intestine (Fig. 1c), forming thicker layers at the base of the epithelium. Posterior intestine from RCPT fish showed a moderate number of Zap70^+^ cells with a more disorganized pattern, appearing also in the lamina propria-submucosa. The amount of Zap70^+^ cells in RCPT posterior intestines was not visibly different from what was observed in CTRL fish (Fig. 1d).

CTRL fish intestines showed scarce and dispersed Tbet^+^ cells, appearing mainly at the base of the epithelium. Positive cells were difficult to locate and no more than one or two cells were present per field of observation (Fig. 1e, f). In RCPT fish intestine, Tbet^+^ cells were visibly more numerous, mainly at the base of the epithelium and between the enterocytes in anterior intestine (Fig. 1g), or also in the lamina propria-submucosa at the posterior intestine (Fig. 1h).

### Zap70^+^ T cells were not actively proliferating at the sampling time

**Fig. 2:**
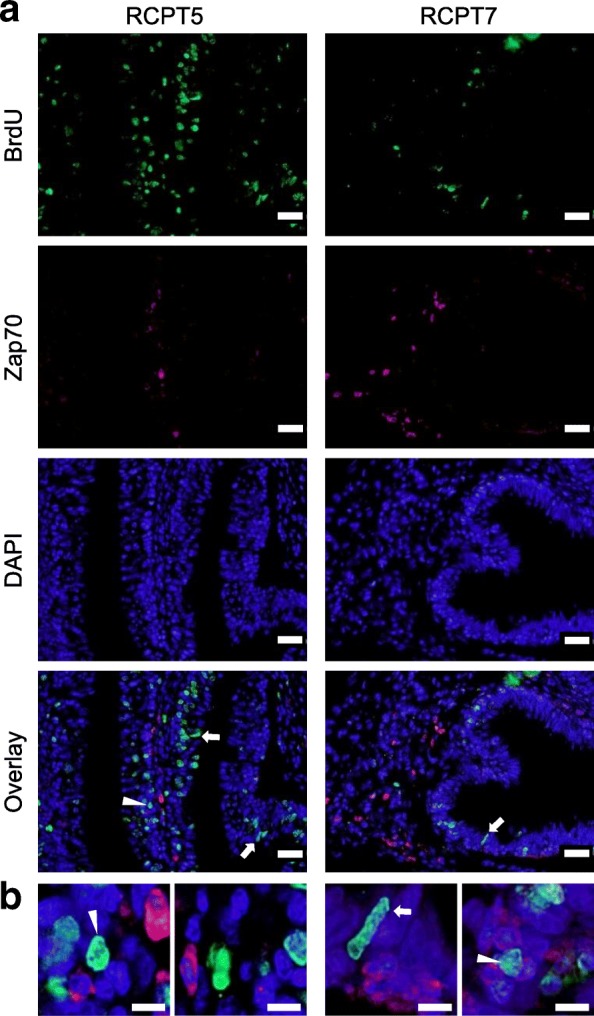
The higher numbers of Zap70^+^ T cells in infected anterior intestine are not actively proliferating. Representative micrographs of infected anterior intestine that showed high *zap*70 gene expression and higher numbers of Zap70^+^ cells. **a** Overview images from two RCPT fish double stained with anti-Zap70 (red) and anti-BrdU (green) antibodies. Nuclear staining was performed with DAPI (blue). White arrows point to proliferating epithelial cells. White arrowheads point to other proliferating nuclei with lymphocyte-like shape. **b** Details showing scattered or grouped Zap70^+^ cells (red) none of which shows BrdU immunoreactive nuclei. Conversely, BrdU^+^ cells did not show Zap70 immunoreactivity. *Scale-bars*: **a**, 20 μm; **b**, 5 μm

RCPT anterior intestines that showed high expression of *zap70* and high abundance of Zap70^+^ cells were further studied to assess whether these higher numbers were due to active proliferation. As expected for this fish-parasite model [26], BrdU positive nuclei were abundant in RCPT anterior intestines. These proliferating cells were mainly located at the epithelium with a morphology corresponding to epithelial cells although some smaller nuclei at the base of the epithelium and even in the lamina propria-submucosa were observed. However, in all of the three fish examined, the nuclei of the Zap70^+^ cells did not show reactivity with the anti-BrdU antibodies, indicating that T cells were not proliferating, at least in the last 24 h prior to the sampling (Fig. 2).

### The expression of T cell signature molecules correlates with infection status in the target organ

**Fig. 3:**
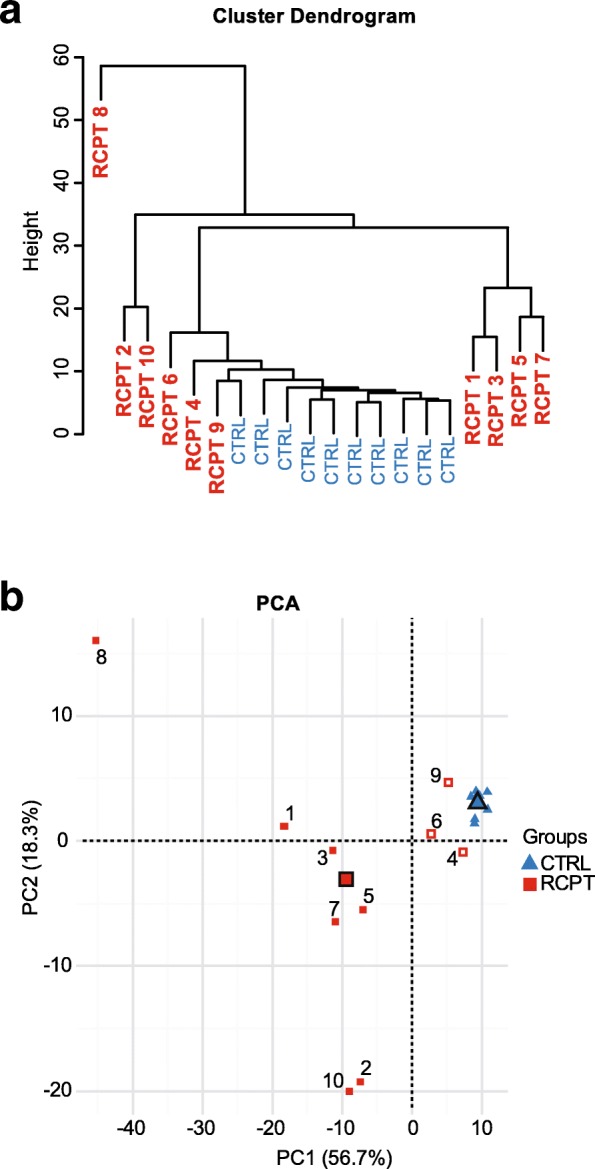
Infection status correlates with differential clustering of recipient fish. **a** Hierarchical cluster analysis using individual expression values of the T cell signature molecules in all tissues of the 10 control (CTRL, blue) and 10 recipient (RCPT, red) fish. RCPT fish numbers correlate with the numbers in Table 2. **b** Principal components analysis constructed using individual expression values of the T cell signature molecules in all tissues of the 10 CTRL (blue triangles) and 10 RCPT (red squares) fish. Numbers correlate with the respective RCPT fish. Bigger symbols represent the centroids for each group. The recipient fish that clustered within the CTRL group in the hierarchical cluster are depicted in red and white squares. Numbers of the individual CTRL fish were removed for clarity

Hierarchical cluster analysis of the expression values for all genes related to T cell responses, considering all organs and individuals, showed that all CTRL fish clustered together, whereas RCPT fish had more variability, clustering in three different branches (Fig. 3a). Three RCPT fish appeared in the CTRL cluster, namely RCPT4, RCPT6 and RCPT9. Interestingly, these three RCPT fish scored 0 for parasite presence in all intestinal segments (RCPT^−^, Table 2). Principal components analysis supported this distinction (Fig. 3b). The centroids of both groups showed a clear separation in the two principal components, which explain in total 75% of the variability. The three RCPT^−^ fish appeared closer to the CTRL cluster and their variability among each other was low.

**Fig. 4:**
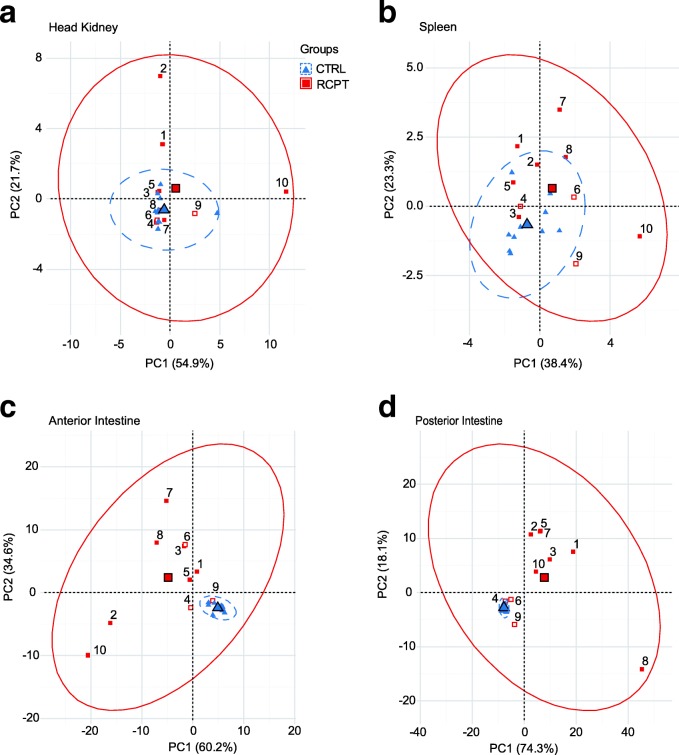
*Enteromyxum leei* exposure elicits changes in the expression of T cell signature genes at the local level. Principal component analyses constructed using individual expression values of the T cell signature molecules in head kidney (**a**), spleen (**b**), anterior (**c**) and posterior intestine (**d**). Numbers correlate with the respective RCPT fish (red squares). Bigger symbols represent the centroids for each group’s ellipse. The recipient fish that clustered within the CTRL group (blue triangles) in Fig. 3a are depicted in red and white squares. Numbers of the individual CTRL fish were removed for clarity

Considering that *E. leei*’s target organ is the intestine and taking into account that the most drastic changes in gene expression were observed in that organ (Table 3), PCA was also performed for each organ separately, to assess whether changes were tissue specific or systemic. Variability in overall expression values of T cell signature molecules in head kidney and spleen of CTRL and RCPT fish was low (Fig. 4a, b). Individual variability was higher than group variability thus, no clear group separation could be observed within lymphohaematopoietic organs. By contrast, in the intestine, the variability of the RCPT group was significantly higher and CTRL and RCPT fish appeared clearly separated (Fig. 4c, d). This effect was more pronounced in the posterior intestine (Fig. 4d), where the different infection status become evident. RCPT^−^ fish clustered close together near the CTRL group evidencing that the highest and more specific response is taking place in this organ and this particular segment.

### Exposed but non-infected fish display an intermediate phenotype between control and infected fish

**Fig. 5:**
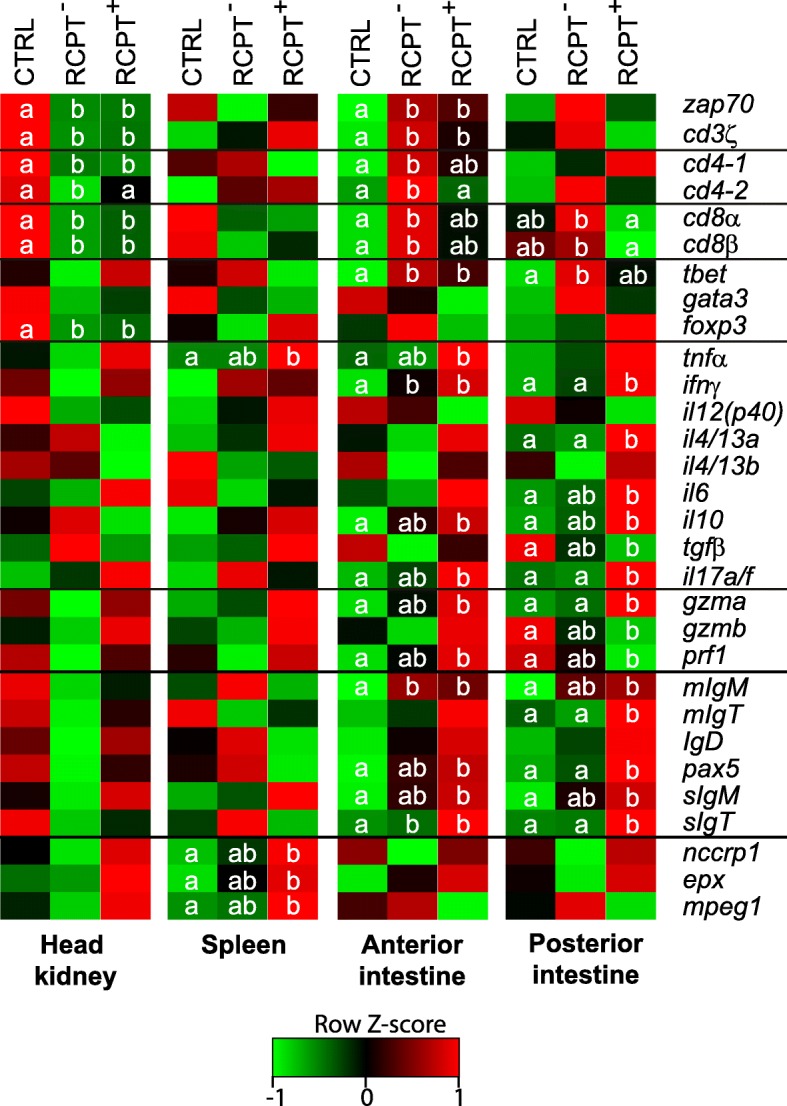
*Enteromyxum leei*-exposed non-infected fish show an intermediate phenotype at a local level. Heatmaps depicting the differential expression of all studied genes in the different tissues. Average expression values of the 10 control unexposed (CTRL) fish, the 3 recipient (RCPT) fish that were negative for the parasite (RCPT^−^) and the 7 RCPT fish that scored positive for the parasite (RCPT^+^) were used to construct the heatmaps. Values are centred and scaled per row. Thus, the different colours show the standard deviations from the mean per row (row Z-score). Different letters indicate statistical differences in expression when comparing the three groups by ANOVA (*P* < 0.05)

A new analysis of the gene expression values was performed, considering three groups, namely CTRL, RCPT^+^ (fish positive for the parasite in at least one intestinal segment) and RCPT^−^ (exposed fish where no parasite was detected). The heatmaps in Fig. 5 show the differential expression of all genes in the three different groups and four tissues. Significant changes in head kidney and spleen were scarce and did not particularly differ from what was observed in the previous analysis (Table 3). In head kidney, RCPT^−^ fish showed significantly downregulated *cd4-2* expression (ANOVA: *F*_(2, 17)_ = 3.43, *P* = 0.05), while in spleen RCPT^−^ always displayed an intermediate phenotype in the differentially regulated genes. In the anterior intestine, RCPT^−^ fish showed a more specific phenotype with upregulation of T_H_ (*cd4-1* (ANOVA: *F*_(2, 17)_ = 8.58, *P* = 0.0027) and *cd4-2* (ANOVA: *F*_(2, 17)_ = 6.083, *P* = 0.0102)) and CTL (*cd8α* (ANOVA: *F*_(2, 17)_ = 4.889, *P* = 0.021) and *cd8β* (ANOVA: *F*_(2, 17)_ = 4.504, *P* = 0.027)) receptors. RCPT^−^ phenotype in posterior intestine was generally intermediate between CTRL and RCPT^+^ groups, but had larger differences with the RCPT^+^ group than with the CTRL group, explaining the clustering closer to the CTRL group in Fig. 4d.

## Discussion

The aim of the current study was to gain insights of the T cell response of gilthead sea bream upon infection with *E. leei*. Previous studies using this host-parasite model showed that *E. leei* modulated different leukocyte populations, both at a local and systemic level. It induced proliferation of leukocytes in head kidney with recruitment into intestines *via* blood circulation, a general increase in mast cells and depletion of acidophilic granulocytes [8]. IgM^+^ B cells and secreted and membrane *IgM* and *IgT* transcripts increased in infected intestine [3, 4, present study]. The current results show an increased number of secreted *IgM* and *IgT* transcripts in the target tissues of infected animals and point to the proliferation of IgM^+^ and IgT^+^ cells, supported by increased numbers of membrane *Ig* transcripts and increased expression of *pax5*, a marker for B cell differentiation [25, 28]. Interestingly, in RCPT^−^ fish, the B cell response was almost equal to that of CTRL fish (Fig. 5). To date, the T cell response in this host-parasite model has not been specifically studied.

**Fig. 6:**
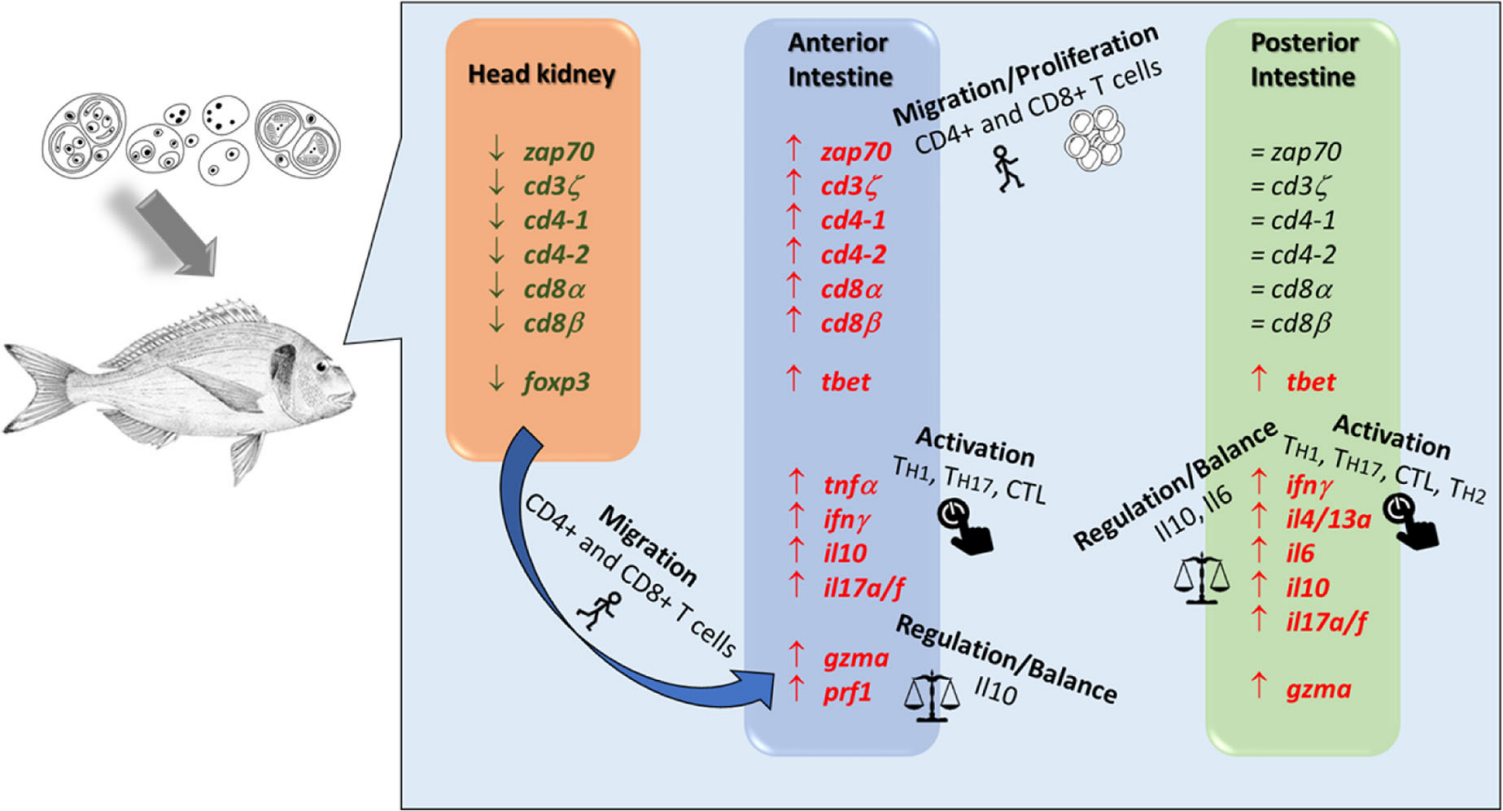
Schematic summary of the PCR-array results on T cell signature molecules showing key signature genes and their expression changes in head kidney, anterior and posterior intestine of gilthead sea bream upon infection with *Enteromyxum leei*, including the hypothesis extracted from the results. Green and red represent significantly down- and upregulated genes, respectively, in RCPT fish. Genes indicated in black showed no significant changes

The study of fish T cell responses has been hampered by the lack of specific antibodies and molecular tools. Nowadays, several T cell specific antibodies have been developed for a few fish species such as rainbow trout (*Oncorhynchus mykiss*), spotted green pufferfish (*Tetraodon nigroviridis*), ginbuna crucian carp (*Carassius auratus langsdorfii*) or Japanese pufferfish (*Takifugu rubripes*) [29–34] and some showed cross-reaction with close species [30]. In addition, CD4^+^ T cell lines have recently been established for common carp [35] and rainbow trout [32] and also CD4-transgenic zebrafish have been created [36]. Recent studies addressing the functional characterization of fish T cells subpopulations, point to a certain degree of conservation with mammals [21, 37]. Regretfully, not many tools are available to study these cell populations outside salmonids and cyprinids. Consequently, we approached our research using transcriptomic tools and validated the observations with the available antibodies, aiming not to infer absolute conclusions, but to obtain hints to base future functional studies. A schematic summary of the results of this study is shown in Fig. 6 to facilitate the global overview of the responses in the different tissues, as discussed below.

Upon activation, the TCR transmits the signal *via* the CD3 complex. In teleost fish, three CD3 chains have been identified, CD3γδ, CD3ε and CD3ζ, the later responsible for the recruitment and activation of the Zap70 kinase [19]. Hence, the CD3 chains and Zap70 are commonly used *pan* T cell markers both in mammals and fish [21, 25]. In the present study, *cd3ζ* and *zap70* expression levels were very consistent, being both up- or downregulated simultaneously. The expression of *zap70* was also confirmed at protein level. High expression values for *zap70* correlated with a higher number of Zap70^+^ cells in gilthead sea bream intestine. Zap70^+^ cells had a morphology and tissue location consistent with intestinal intraepithelial T lymphocytes. *E. leei* exposure induced *zap70* downregulation in head kidney and an upregulation in anterior intestine. The lower expression of *cd4-1*, *cd4-2*, *cd8α* and *cd8β* in head kidney and the increased expression of the same genes in anterior intestine indicate that both T_H_ cells and CTLs were modulated during the infection. Of note, the downregulation in head kidney was characterized by changes in *pan* T cell markers, *cd4* and *cd8* expression, whereas the expression of the effector cytokines and enzymes did not change. Changes in specific markers of effector phenotypes were only detected in the target tissue, where the parasite is located.

The increased expression of *zap70* and higher number of Zap70^+^ cells in infected anterior intestine can be due to migration from lymphohaematopoietic tissues, local proliferation of T cells, or both. The reverse trend of expression of *pan* T cell markers and receptors between head kidney and anterior intestine in infected fish seems to indicate migration. However, further functional studies should be conducted to verify this hypothesis. Adoptive cell transfer has been successfully used in T cell studies in teleosts [38, 39], but no clonal lines of gilthead sea bream are available. On the other hand, an in vivo proliferation assay on gilthead sea bream intestine by the use of BrdU immunostaining has recently been optimized in our group [26]. This study showed that, upon infection with *E. leei*, intestinal epithelial cells and lymphocyte-like cells showed increased proliferation when compared to control fish. However, in the current study, when double staining with the Zap70 antibody was performed, none of the Zap70^+^ cells had a BrdU immunoreactive nucleus. These results indicate that, at least in the last 24 hours, intestinal Zap70^+^ cells were not proliferating. Thus, with the current tools available for gilthead sea bream, we have more evidence pointing towards migration of T cells to the target organ than to *in situ* proliferation. Time-course studies and migration assays should be conducted in the future to solve this question.

Teleosts have two *cd4* genes that are structural orthologues of the mammalian *CD4*. Teleost *cd4-1* has four Ig domains like the one from tetrapods, while *cd4-2* has two Ig domains and is believed to be a reminiscent of the primordial gene that gave rise to *cd4* and *lag-3* by tandem duplication. Both *cd4* genes contain the canonical Lck association motif essential for the initiation of the T cell activation cascade [19, 40, 41]. In rainbow trout, most T_H_ lymphocytes showed co-expression of both *cd4-1* and *cd4-2* in their membrane, while a small uncharacterized lymphocyte population appeared *cd4-2* single positive [29]. The same study showed that a significant myeloid population expressed *cd4-1*, just like monocyte/macrophages from rats and humans [42]. Thus, the expression of the four-domain *cd4-1* gene should not be directly linked to T_H_ responses in the absence of other markers. The results in the current study show a parallel regulation of *cd4-1* and *cd4-2* in head kidney (downregulation) and anterior intestine (upregulation) which, together with the *pan* T cell markers information and unresponsiveness of the macrophage specific *mpeg-1* [43], indicate that *E. leei* is regulating T_H_ responses in these organs. The highest expression of both *cd4* genes in anterior intestine was found in exposed, but non-infected fish (RCPT^−^), pointing towards a higher number of T_H_ cells in these animals.

When naïve CD4^+^ T cells become activated, they proliferate and differentiate into distinct T_H_ subsets [44]. Each subset expresses a battery of signature genes that can provide important insights on the populations. However, the expression of certain groups of genes is not definitive due to overlapping. Different leukocytes, and even different T cell subsets, can express certain genes in common. Therefore, differences are better assessed by up- or downregulation of a battery of genes, more than by restricted expression of single molecules [45]. In mammals, T_H1_ activation is initiated by IL12 and IFNγ, Tbet being the master regulator of this subset. *Tbet* expression is not exclusive for T_H1_ cells, it is also expressed in IFNγ stimulated monocytes and dendritic cells, B cells, NK cells and CTLs. Unlike Tbet, the T_H2_ master regulator GATA3 is already expressed in naïve CD4^+^ T cells [16, 46, 47]. Mucosal tissues, due to their constant exposure to external antigens, have been described as T_H2_-skewed. Mucosal regulation of T_H2_ responses is key to maintain balance of host immune homeostasis and defence against pathogens [48]. In fish mucosal tissues, the relative expression of *gata3* is higher than that of *tbet* [49, 50]. However, when considering the ratio of the effector molecules (*il4/13a* and *ifnγ*) trout gills and skin, but not intestines, showed this T_H2_ skewing [51]. In the current study, the ratio T_H1_ / T_H2_ in CTRL fish, calculated in two different ways (*tbet* / *gata3* and *ifnγ* / *il4/13a*) [51, 52], showed that T_H1_ profiles were more dominant in head kidney and spleen, whereas intestine was more skewed to the prototypical T_H2_ profile of mucosal tissues. Thus, in our model, gilthead sea bream intestines clearly showed this T_H2_ profile typically related to mucosal tissues.

The current results suggest that the increase in T_H_ cells induced by the parasitic infection in gilthead sea bream anterior intestine is due to activation of T_H1_ and T_H17_ profiles. This is supported by the significant upregulation of *tbet*, *ifnγ*, and *il17a*/*f*, and the higher number of Tbet^+^ cells. In posterior intestine, a mixed profile with the additional upregulation of *il6* and *il4/13a* (T_H2_) was detected. However, in RCPT posterior intestine upregulation of *pan* T cell markers, *cd4* or *cd8* was not observed. One explanation for this could be that the response in this segment is not characterized by increased cell numbers nor proliferation, but more by activation of resident cells, as opposed to the hypothesized migration in anterior intestines discussed above. In rainbow trout, infections with the myxozoan parasite *Tetracapsuloides bryosalmonae* induced an increase of *tbet* and *ifnγ* expression in the kidney, the target organ for this parasite. In the same study, almost no change was detected for *gata3* [49]. A similar study in *T. bryosalmonae*-infected trout, showed upregulation of *il10*, *il6*, *ifnγ*, *tbet* and T_H17_ cytokines in trunk kidney [53].

Mammalian IL17A and IL17F, and the fish homologue, Il17a/f, can be expressed by many cells, but their major producers in vivo are T_H17_ cells [20]. Our results show that *il17a*/*f* is among the most strongly upregulated genes in parasitized intestines. This is in accordance with what was detected in common carp during infections with the blood parasite *Trypanosoma carassii*, which induced a T_H17_ profile with upregulation of *il17a/f2* [54]. In mammals, IL6 is known to have a pivotal role in switching the immune response from tolerance to inflammation by increasing the expression of *RORγt* (T_H17_) and inhibiting *FOXP3* (T_reg_). IL6 skews the T_H1_ / T_H2_ balance towards T_H2_ by directly inducing expression of *IL4* [55]. However, some studies showed that both IL6 and TGFβ are required for T_H17_ development [56]. In the current study, *il6* was upregulated only in the posterior intestine, whereas *il17a/f* was upregulated in both intestinal segments. This higher expression of *il6* in the posterior intestine was concurrent with a higher expression of *il4/13a* and downregulation of *tgfβ* supporting a role for Il6 in T_H2_ development in this model.

Mammalian T_regs_ are CD4^+^/CD25^+^/FOXP3^+^ cells with immunosuppressive activities mainly due to their production of the anti-inflammatory cytokines TGFβ and IL10 [18]. Immunosuppressive CD4-2^+^/CD25^+^/Foxp3^+^ cells have been functionally characterized in tetraodon, demonstrating the existence of T_reg_ homologues in teleosts [34]. The expression profiles found in our study do not support major changes in the T_reg_ population, although the expression of *il10* was clearly induced in spleen and intestine of RCPT fish. IL10 is a pleiotropic regulatory cytokine that can be produced by many different cell types and is crucial to maintain intestinal tolerance [57]. In fact, different common carp isolated leukocyte populations showed the ability to express *il10* [58]. Thus, the increased expression of *il10* detected in our study is most likely due to a different cell population than T_regs_ and is probably aimed to control the damage that excessive inflammation can cause to host tissues. Upregulation of *il10* and *il6* has already been described in the posterior intestine of *E. leei*-infected gilthead sea bream [9]. The latter study linked the upregulation of these two genes to an anti-inflammatory profile, although the expression of *ifnγ* and *il17a/f*, pro-inflammatory cytokines significantly upregulated in the current study, was not studied then. The expression of *il10* and *il6* was significantly upregulated together with *ifnγ*, *il17a*/*f* and *il4/13a* in RCPT^+^ fish intestines. In RCPT^−^ (exposed, non-infected) fish, these cytokines returned to control expression levels. These new findings indicate that the increased expression of *il6* and *il10* are more likely linked to limit, but maintain, an inflammatory response against the parasite. These results corroborate previous findings in other myxozoan infections, where a dysregulated T cell response characterized by T_H1_ and T_H17_ profiles, together with upregulation of anti-inflammatory/regulatory cytokines (*il10* and *il6*) and increased expression of immunoglobulins (mainly secreted) was found [53]. Of note, although the latter and present studies used different fish species and different myxozoan parasites with different target tissues, both models elicited very similar responses.

The prototypical cytotoxic activity of mammalian CTLs has also been described in fish [59–62]. Similar to what was found for T_H_ cells, our results showed upregulation of both *cd8* genes in the anterior intestine, which correlated with downregulation in head kidney, upon infection. CTL activity in *E. leei*-infected anterior intestine was supported by a significant increase in *gzma* and *prf1* expression. In all vertebrates, CTLs and NK cells are known to express several receptors, transcription factors and effector molecules in common [63]. Nevertheless, our results show that the increased *cd8* expression detected is not correlated with changes in expression of *nccrp1*, a receptor needed for recognition of target cells expressed in natural cytotoxic cells (NCC), the teleost NK equivalents [64]. CTLs also express Tbet and IFNγ [13], thus the increased expression of these molecules can also be attributed to such cell population. In any case, *E. leei* infection clearly induced a type 1 immune response in the target tissue. Activated teleost CTLs have been described to be major producers of Ifn*γ*, granzymes and perforin [65]. In the current study, *gzma* was one of the most upregulated genes in both intestinal segments upon *E. leei* infection; and while *prf1* showed the same trend in anterior intestine, *gzmb* did not. Mammalian studies on the dynamics of expression of effector molecules in CTLs showed that GzmA^+^/GzmB^−^ cells are detected earlier than GzmA^+^/GzmB^+^ cells. In addition, GzmA has trypsin like activity cleaving after the basic residues arginine and lysine, whereas GzmB cleaves after aspartic acid residues [66]. Thus, the expression profile detected in our study could indicate (i) a time-point situation where only Gzma^+^/Gzmb^−^ cells are present; (ii) a specific regulation where only trypsin like activity is required; or (iii) a dysregulation of the CTL response induced by the parasite. Of note, RCPT^−^ fish showed higher levels of *cd8* expression in intestine, whereas the expression of the effector molecules was only higher in RCPT^+^ fish. Thus, we hypothesize that fish with more CD8^+^ cells or with higher levels of activation of this subtype have more chances to successfully avoid or clear the infection. A recent study in Atlantic salmon (*Salmo salar*) infected with the myxozoan *Kudoa thyrsites* showed that CTLs are key for resolution of infection and protection against reinfection [67].

CTLs are classically considered to specifically target virus-infected, tumor and allogenic cells. However, recent studies demonstrated that their role expands to the ability of directly killing microbial pathogens, including extracellular parasites [68]. In fact, cell cytotoxic activity has already been described as one of the main immune mechanisms involved in gilthead sea bream immune response to *E. leei* [69]. The possibility that the infection method used (anal intubation of infected intestinal scrapings) could elicit a CTL reaction against the allogenic cells present in the inoculum could raise some concern. In common carp, anal administration of allogenic cells induced specific cell-mediated cytotoxicity which peaked seven days after immunization but, in the absence of re-exposure to the antigen, returned to control levels after 20 days [70]. Our observations, performed ten weeks after inoculation, should only be due to the parasitic infection, which actually establishes and progresses in the intestine, while the allogenic cells are rapidly eliminated. Supporting this, infections by cohabitation with *E. leei-*infected fish, also elicited cytotoxic cell activity [69].

Taken together, the analysis of the expression levels of the genes selected for this study effectively separated infected (RCPT^+^) from non-infected (RCPT^−^ and CTRL) fish. It is a fact that not one immune response is uniquely represented by a single T cell subset, but a particular phenotype could be overrepresented. *E. leei* seems to have induced the migration of T cells from head kidney to the anterior intestine and type 1 (T_H1_ and CTL) and T_H17_ profiles in both intestinal segments. The higher expression of regulatory cytokines is probably balancing these inflammatory profiles to avoid damage (Fig. 6). By using the anal intubation route of infection, we assured that all RCPT fish were exposed to similar amounts of parasite at the same time. Moreover, all individuals from the RCPT group showed infection signs based on their biometrical parameters. This indicates that the 30% of fish that were negative for the parasite at the final sampling (RCPT^−^), had successfully avoided parasite infection or had recovered from an initially established infection that did not progress. Interestingly, the expression profile of these exposed, but non-infected fish was more similar to the control than to the infected fish. A similar intermediate phenotype has been previously described using a massive analysis approach [71], supporting the current findings. This was more evident at the intestinal level, particularly in the posterior intestine, and we hypothesize that an effective and regulated CTL response is essential for the clearance of the parasite. Differential gene expression profiles between RCPT^+^ and RCPT^−^ fish, particularly in the intestine, have already been described for this infection model [71, 72]. One of these studies showed that Ifn-stimulated genes were specifically upregulated in RCPT^−^ animals [71], but the expression of *ifnγ* was never directly measured. Collectively, all results indicate that a type 1 response at the site of infection is needed to face and overcome this parasite.

There are still many unexplored facts to be addressed in future studies. For example, the sequences for *il2*, important T cell growth factor, or *il21* and *rorγt*, effector cytokine and master regulator of T_H17_ responses [20], were not found in the currently available genomic and transcriptomic data of gilthead sea bream and were consequently not studied. In addition, the existence of different isoforms for duplicated genes, so common in teleosts [73], was not detected with the current genomic data, but should not be discarded until more information is available. For instance, in the evolutionarily close European sea bass, two *il4/13a* isoforms have been described [74], and our preliminary analyses point to a similar event in gilthead sea bream that requires further study. The development of specific antibodies is also crucial to precisely define cell subtypes and functionally characterize them. Nonetheless, this study provides important clues on the gilthead sea bream intestinal T cell responses and can serve as a basis to better understand the response against this important parasite in order to establish effective preventive or palliative measures.

## Conclusions

The results of the present study evidence a clear modulation of T cell responses in gilthead sea bream upon infection with *E. leei*. T cells appear to migrate from head kidney to the site of infection where they become activated. Type 1 responses are the most represented at a local level and the high expression of regulatory cytokines keeps this inflammatory profile in balance. The global analysis of these T cell markers in the intestine can provide information of their infection status.

## Additional files

Additional file 1: **Table S1.** Primers used in the study. (PDF 458 kb)

Additional file 2: **Table S2.** Gene expression data relative to *β-actin* for each individual control (CTRL) and recipient (RCPT) fish used in the study. (PDF 501 kb)

## Abbreviations

BrdU: Bromodeoxyuridine
CTL: Cytotoxic T lymphocyte
CTRL: Control/unexposed
Gzm: Granzyme
Ifn: Interferon
Ig: Immunoglobulin
IL: Interleukin
NCC: Non-specific cytotoxic cell
NK: Natural killer
p.i.: Post-intubation
RCPT: Recipient/exposed
RCPT^−^: Recipient negative for parasite presence
RCPT^+^: Recipient positive for parasite presence
SEM: Standard error of the mean
TCR: T cell receptor
TGFβ: Transforming growth factor beta
T_H_: Helper T cell
T_reg_: Regulatory T cell
