# Supplementary material for: Hints on T cell responses in a fish-parasite model: Enteromyxum leei induces differential expression of T cell signature molecules depending on the organ and the infection status
Source: Parasit Vectors. 2018 Jul 31;11:443. doi: 10.1186/s13071-018-3007-1 (PMC6069777; doi:10.1186/s13071-018-3007-1)
Supplement: Supplementary file 1 — Table S1. Primers used in the study. (PDF 458 kb) [file 13071_2018_3007_MOESM1_ESM.pdf]

Additional File 1: Table S1. Primers used in this study.

Gene name	Symbol	Accession Number		Sequence (5'-3')
β actin	*actβ*	X89920	F	TCCTGCGGAATCCATGAGA
			R	GACGTCGCACTTCATGATGCT
Zeta-chain-associated protein kinase 70*	*zap70*	MF175239	F	TGGTGAAGGAGGAGATGATGAGG
			R	GCGAACGATGTAGCGGTTGT
Cluster of differentiation 3 zeta chain*	*cd3ζ*	MF175235	F	ATGGCGGTCCAGACGAGGGTTTC
			R	ACCAGCGAGGACAGGACCAGCAG
Cluster of differentiation 4-1	*cd4-1*	AM489485	F	TCCTCCTCCTCGTCCTCGTT
			R	GGTGTCTCATCTTCCGCTGTCT
Cluster of differentiation 4-2 (rel)*	*cd4-2*	MF175230	F	GTGGTGGATGTGGGTTGTAATTGGAGTT
			R	AAGACAATGACAAGGACCATCAGGAGGA
Cluster of differentiation 8 alpha	*cd8α*	*EU921630*	F	GCAGCAACGGTAACACGAACG
			R	CCAGTATGAGCGGAGTACAGAACA
Cluster of differentiation 8 beta	*cd8β*	*KX231275*	F	CCGAAATGTGGAAGACTGGAACTC
			R	CTTTGGAGGTAAGGTTGGAGGGAT
T box transcription factor TBX21*	*tbet*	MF175241	F	GCACCCCGACTCTCCCAACA
			R	GCTGAAAGACACTTCTTGCCTCATCCA
Trans-acting T-cell-specific transcription factor GATA3	*gata3*	KU302761	F	CCTCCGTACTATGGAAACTCTG
			R	ACCTGGCTACTGTGTGGA
Forkhead box P3*	*foxp3*	MF175231	F	TTCAGACACAACACCGCAACCT
			R	TCTGTCCACTCGCACAAAGCATTT
Tumor necrosis factor alpha	*tnfα*	AJ413189	F	CAGGCGTCGTTCAGAGTCTC
			R	CTGTGGCTGAGAGCTGTGAG
Interferon gamma*	*ifnγ*	MF175242	F	CTGATTCTCATGGTGGCTCTGT
			R	CGCAGGAGGCTCTGGATG
Interleukin 12 (p40 subunit)	*il12(p40)*	JX976624	F	ATTCCCTGTGTGGTGGCTGCT
			R	GCTGGCATCCTGGCACTGAAT
Interleukin 4/13 a*	*il4/13a*	MG816479 [1]	F	GCTTCTTCTGGTGTCCGCTGTG
			R	AGACGCCTGAATGTGAGCAAGACT
Interleukin 4/13 b*	*il4/13b*	MG816480 [2]	F	GCTGAGAAGTCCCTGGAAGCACACAATA
			R	TACCGACACAAGTGACGAGTAAGGTTTGAT
Interleukin 6	*il6*	EU244588	F	TCTTGAAGGTGGTGCTGGAAGTG
			R	AAGGACAATCTGCTGGAAGTGAGG
Interleukin 10	*il10*	JX976621	F	AACATCCTGGGCTTCTATCTG
			R	GTGTCCTCCGTCTCATCTG
Transforming growth factor beta	*tgfβ*	AF424703	F	GGAGATTACCGCCTGCTGAC
			R	CCGCTGCTCATCAAGTATCGT
Interleukin 17 a/f*	*il17a/f*	MF175237	F	CCTCTGATGGTGCGGTGATAG
			R	CCTGATGGGTTTGCTGGGAAC
Granzyme a*	*gzm a*	MF175243	F	GCCCTGATGTCTGCCAATGTGA
			R	GCCCTCCTGAGTCCCCCTG
Granzyme b*	*gzm b*	MF175232	F	CCAGAGAGGCAGGAGGGATATG
			R	AGGCTTGGTGTTGCAGATGAG
Perforin 1*	*prf1*	MF175233	F	ACGGATGGCTATGTGAAGGTGTC
			R	TGTGGGTTGTTGTTGTTGTGAATGA
Immunoglobulin M (membrane)	*mIgM*	KX599199	F	GCTATGGAGGCGGAGGAAGATAACA
			R	GCAGAGTGATGAGGAAGAGAAGGATGAA
Immunoglobulin T (membrane)	*mIgT*	KX599201	F	AGACGATGCCAGTGAAGAGGATGAGT
			R	CGAAGGAGGAGGCTGTGGACCA
Immunoglobulin D*	*IgD*	MF974245	F	GACCCACAGCAAACCTTCAG
			R	AGAGACCAGGCACACGATT
Paired box protein Pax5*	*pax5*	MF175229	F	GGCTCCTCGTACTCCATCAG
			R	TCTTCCTCTTGCCGACATCAG
Immunoglobulin M (secreted)	*sIgM*	JQ811851	F	ACCTCAGCGTCCTTCAGTGTTTATGATGCC
			R	CAGCGTCGTCGTCAACAAGCCAAGC
Immunoglobulin T (secreted)	*sIgT*	KX599200	F	GCTGTCAAGGTGGCCCCAAAAG
			R	CAACATTCATGCGAGTTACCCTTGGC
Nonspecific cytotoxic cell receptor protein-1	*nccrp1*	AY651258	F	AGGTGTGAGGCTGAGTGGAGTCTG
			R	AGTAGATTCCTCCCGAGCGGTTTGG
Eosinophil peroxidase*	*epx*	MF175227	F	CAGCCCGATGGAGGTGAA
			R	GCGTTGGAGTCGTTGGTG
Macrophage expresed gene 1*	*mpeg1*	MF175228	F	GCCAGACAACTGAAGACGGACTCTACCT
			R	TGCGGGACGACAAACACCTCATCT

Asterisks (*) indicate sequences described for the first time in gilthead sea bream.

[1,2] Sequences found by analysis of the draft gilthead sea bream genome (http://nutrigroup-iats.org/seabreamdb/). [1] *il4/13a* was identified by synteny analysis as an open reading frame between *pou4f3* and *rad50*; and [2] *il4/13b* appeared adjacent to *kif3a*. Both sequences showed 47% and 53% identity with sea bass *il4/13a* and *il4/13b* respectively [74].
