# Supplementary material for: Hints on T cell responses in a fish-parasite model: Enteromyxum leei induces differential expression of T cell signature molecules depending on the organ and the infection status
Source: Parasit Vectors. 2018 Jul 31;11:443. doi: 10.1186/s13071-018-3007-1 (PMC6069777; doi:10.1186/s13071-018-3007-1)
Supplement: Supplementary file 2 — Table S2. Gene expression data relative to β-actin for each individual control (CTRL) and recipient (RCPT) fish used in the study. (PDF 501 kb) [file 13071_2018_3007_MOESM2_ESM.pdf]

Additional File 2: Table S2. Gene expression data relative to *β-actin* for each individual control (CTRL) and recipient (RCPT) fish used in the study.

Head kidney	CTRL1	CTRL2	CTRL3	CTRL4	CTRL5	CTRL6	CTRL7	CTRL8	CTRL9	CTRL10
zap70	0,00165	0,00115	0,00112	0,00131	0,00152	0,00141	0,00112	0,00128	0,00143	0,00124
cd3ζ	0,003217	0,003472	0,002879	0,002472	0,003151	0,00324	0,00294	0,002781	0,003285	0,002801
cd4-1	0,001677	0,000997	0,000804	0,000766	0,00113	0,001169	0,000793	0,000761	0,001219	0,00093
cd4-2	0,001586	0,001772	0,001039	0,001245	0,001219	0,0017	0,00142	0,00143	0,001262	0,00128
cd8α	0,002064	0,001316	0,001391	0,00144	0,00198	0,001381	0,002489	0,000605	0,000892	0,001069
cd8β	0,000357	0,000509	0,000318	0,000425	0,000653	0,000383	0,000569	0,000179	0,000244	0,000269
tbet	0,00292	0,003129	0,004044	0,003086	0,004016	0,003354	0,00296	0,001926	0,002108	0,002781
gata3	0,000799	0,000644	0,0007	0,000804	0,001153	0,000672	0,00061	0,000691	0,002259	0,00073
foxp3	0,000456	0,000242	0,000324	0,000499	0,000506	0,000258	0,000431	0,000309	0,000348	0,000419
tnfα	0,000135	7,73E-05	0,000109	0,000109	0,000125	8,28E-05	9E-05	0,000152	7,78E-05	7,51E-05
ifnγ	4,29E-05	4,66E-05	6,19E-05	4,5E-05	6,68E-05	8,63E-05	4,96E-05	6,73E-05	6,36E-05	7,31E-05
il12 (p40)	2,08E-05	2,39E-05	2,14E-05	1,68E-05	1,38E-05	4,41E-05	1,56E-05	1,1E-05	0,000214	1,81E-05
il4/13a	6,77E-05	5,46E-05	3,19E-06	3,16E-05	6,45E-05	2,35E-05	8,82E-06	2,28E-06	2,64E-05	2,69E-05
il4/13b	0,000459	0,000667	0,000311	0,000383	0,000219	0,000622	0,000334	0,000509	0,00097	0,000365
il6	1,66E-05	5,21E-06	3,53E-05	9,46E-06	1,38E-05	2,48E-05	1,38E-05	2,69E-05	6,07E-06	3,01E-06
il10	0,00068	0,00049	0,00054	0,0009	0,00071	0,00052	0,0008	0,00083	0,00053	0,00066
tgfβ	9,19E-05	5,5E-05	5,98E-05	5,85E-05	5,5E-05	7,41E-05	5,77E-05	8,57E-05	4,96E-05	0,000149
il17a/f	1,29E-06	2,16E-06	2,83E-06	2,85E-05	1,36E-06	2,55E-06	1,67E-06	1,17E-06	2,1E-06	1,48E-06
gzma	5,77E-05	3,14E-06	2,64E-05	1,42E-05	8,06E-06	3,12E-06	1,96E-05	3,61E-06	4,15E-06	1,89E-05
gzmb	0,001353	0,000667	0,001522	0,001631	0,002036	0,000635	0,000983	0,000799	0,001114	0,001061
prf1	0,001084	0,000459	0,0007	0,000523	0,001004	0,000534	0,00085	0,000465	0,000597	0,000557
mIgM	0,028956	0,021051	0,011924	0,021197	0,02977	0,013697	0,015303	0,008729	0,01038	0,015953
mIgT	0,002781	0,00942	0,006801	0,004678	0,007652	0,003044	0,004187	0,008032	0,004364	0,00324
IgD	0,011281	0,014279	0,005226	0,010598	0,013508	0,00367	0,008669	0,01038	0,007239	0,017217
pax5	0,00639	0,005263	0,004395	0,006801	0,010309	0,004364	0,006302	0,006172	0,004581	0,00568
sIgM	0,312083	0,104386	0,160428	0,05366	0,386891	0,144586	0,14161	0,069348	0,148651	0,094732
sIgT	0,000251	3,74E-06	2,25E-06	0,00019	0,000459	2,74E-06	1,99E-06	0,000534	0,000462	1,84E-06
nccrp1	0,04181	0,022406	0,031034	0,036651	0,035158	0,034674	0,032129	0,05329	0,037163	0,051474
epx1	0,057114	0,038741	0,031686	0,055169	0,048698	0,063813	0,057912	0,08362	0,0625	0,096055
mpeg1	0,004016	0,003217	0,003354	0,005013	0,004245	0,004364	0,003448	0,003023	0,00286	0,005839

Head kidney	RCPT1	RCPT2	RCPT3	RCPT4	RCPT5	RCPT6	RCPT7	RCPT8	RCPT9	RCPT10
zap70	0,00108	0,00097	0,00056	0,00083	0,00071	0,00091	0,001	0,0008	0,00073	0,00078
cd3ζ	0,001362	0,002743	0,001076	0,001913	0,00282	0,001926	0,001785	0,001822	0,001642	0,0017
cd4-1	0,000318	0,00081	0,000402	0,000557	0,000449	0,000653	0,00052	0,000475	0,00037	0,000601
cd4-2	0,000793	0,001597	0,000523	0,000997	0,00093	0,000788	0,000793	0,00075	0,000499	0,002152
cd8α	0,000492	0,001236	0,000691	0,000618	0,001039	0,001039	0,000983	0,000553	0,000419	0,000691
cd8β	5,66E-05	0,000208	0,000182	0,000175	0,00037	0,000233	0,000265	0,000137	7,31E-05	0,000141
tbet	0,001953	0,004979	0,002743	0,002355	0,003988	0,00294	0,002152	0,004158	0,001511	0,004456
gata3	0,000627	0,000793	0,000899	0,000905	0,000777	0,000402	0,000513	0,000681	0,000452	0,000452
foxp3	0,000176	0,000413	0,000168	0,000189	0,000205	0,000226	0,00028	0,000184	0,000216	0,000175
tnfα	7,73E-05	0,000127	9E-05	0,000138	0,000105	3,14E-05	0,000275	6,19E-05	0,000122	4,5E-05
ifnγ	7,11E-05	7,89E-05	5,03E-05	3,14E-05	5,31E-05	4E-05	4,92E-05	5,28E-05	5,58E-05	7,89E-05
il12 (p40)	1,53E-05	2,81E-05	1,06E-05	1,47E-05	1,61E-05	2,16E-05	3,16E-05	2,19E-05	1,14E-05	2,6E-05
il4/13a	1,62E-05	2,53E-05	3,43E-05	5,81E-05	3,34E-05	3,71E-05	5,58E-05	9,46E-06	3,14E-06	1,52E-05
il4/13b	0,000348	0,000446	0,000233	0,000462	0,00061	0,000459	0,000509	0,00036	0,000502	0,000492
il6	7,11E-05	0,000133	3,01E-05	4,29E-06	3,01E-05	5,21E-06	7,32E-06	1,17E-05	7,84E-06	2,36E-05
il10	0,0006	0,00072	0,00053	0,00055	0,00058	0,00103	0,00046	0,0004	0,00073	0,00051
tgfβ	6,23E-05	9,19E-05	7,62E-05	0,00016	7,46E-05	3,73E-05	8,87E-05	4,03E-05	7,57E-05	6,45E-05
il17a/f	3,89E-06	3,63E-06	2,13E-06	1,1E-06	1,78E-06	9,6E-07	4,38E-06	2,13E-06	1,78E-05	5,94E-05
gzma	2,5E-05	4,96E-06	1,09E-05	1,03E-05	3,48E-05	1,72E-05	1,21E-05	1,72E-05	3,74E-06	9,39E-06
gzmb	0,001543	0,001018	0,001069	0,000725	0,000905	0,000614	0,003151	0,000899	0,001597	0,001797
prf1	0,000468	0,000618	0,000527	0,000495	0,000658	0,000431	0,001228	0,000225	0,000635	0,000761
mIgM	0,008974	0,028557	0,017579	0,011203	0,015198	0,019915	0,009163	0,015517	0,007652	0,00942
mIgT	0,005013	0,006524	0,004304	0,002801	0,003988	0,001926	0,004016	0,00455	0,001953	0,001654
IgD	0,005486	0,010453	0,010097	0,00564	0,013888	0,011203	0,018073	0,00879	0,008912	0,006434
pax5	0,002801	0,008974	0,005839	0,003906	0,007599	0,006524	0,005299	0,006524	0,005226	0,003308
sIgM	0,234881	0,15932	0,358489	0,184284	0,198884	0,148651	0,105843	0,136787	0,044811	0,124137
sIgT	2,7E-06	1,37E-06	1,74E-06	5,21E-06	0,000365	0,000136	9,14E-06	4,32E-06	3,92E-06	0,000309
nccrp1	0,055939	0,062935	0,047039	0,034674	0,049721	0,024689	0,02836	0,03901	0,027776	0,037944
epx1	0,048361	0,063813	0,083043	0,064257	0,092783	0,057912	0,047366	0,0625	0,05366	0,076415
mpeg1	0,003173	0,006848	0,003377	0,003424	0,006346	0,002577	0,00324	0,003065	0,003594	0,009486

Additional File 2: Table S2. Continued.

Spleen	CTRL1	CTRL2	CTRL3	CTRL4	CTRL5	CTRL6	CTRL7	CTRL8	CTRL9	CTRL10
zap70	0,00134	0,0016	0,00087	0,00111	0,00135	0,00116	0,00224	0,00165	0,00143	0,00124
cd3ζ	0,004876	0,004364	0,003331	0,002438	0,002762	0,00367	0,003472	0,003448	0,006258	0,004334
cd4-1	0,000662	0,00143	0,000622	0,000459	0,000523	0,000534	0,000892	0,000627	0,000618	0,000635
cd4-2	0,001586	0,001772	0,002137	0,00143	0,000695	0,00205	0,0017	0,001712	0,002137	0,002307
cd8α	0,00073	0,002355	0,001712	0,000667	0,001114	0,001018	0,00194	0,001236	0,001298	0,001091
cd8β	0,000157	0,000397	0,000248	0,000112	0,000255	0,000213	0,00028	0,000194	0,000208	0,000167
tbet	0,00282	0,00849	0,003308	0,002631	0,003496	0,00282	0,004487	0,003285	0,003401	0,003331
gata3	0,000886	0,001654	0,000899	0,001236	0,001178	0,003773	0,002064	0,001772	0,002472	0,001543
foxp3	0,000251	0,000198	0,000163	0,000144	0,000233	0,000185	0,000277	0,000284	0,000267	0,000394
tnfα	7,46E-05	6,82E-05	6,91E-05	7,21E-05	0,000136	0,000121	5,31E-05	0,000219	0,000144	0,000217
ifnγ	0,000223	0,000219	0,000161	0,000242	0,000156	0,000175	0,000181	0,000329	0,000327	0,000322
il12 (p40)	2,97E-05	1,69E-05	2,2E-05	3,14E-05	2,1E-05	3,34E-05	2,3E-05	8,05E-05	8,69E-05	9,71E-05
il4/13a	5,1E-05	2,01E-05	2,83E-06	3,2E-05	1,22E-05	1,55E-05	9,01E-06	2,39E-05	8,89E-06	3,51E-05
il4/13b	0,000411	0,000408	0,000207	0,000394	0,000375	0,000284	0,000631	0,000614	0,000294	0,000298
il6	1,26E-05	1,38E-05	1,15E-05	7,52E-06	1,87E-05	1,99E-05	4,05E-05	2,89E-05	1,41E-05	1,53E-05
il10	0,00014	0,00016	0,0001	0,00013	0,00009	0,00013	0,00017	0,00016	0,00015	0,00011
tgfβ	0,000112	6,77E-05	7,67E-05	0,000106	0,000233	8,57E-05	4,99E-05	9,92E-05	7,89E-05	0,00012
il17a/f	1,03E-06	2,09E-06	1,13E-06	2,19E-05	4,54E-06	1,13E-06	2,91E-06	1,04E-05	8,82E-06	9,66E-06
gzma	0,000148	2,89E-05	3,07E-05	6,06E-05	5,39E-05	1,3E-05	2,06E-05	4,69E-05	3,53E-05	5,58E-05
gzmb	0,00162	0,0014	0,001178	0,00149	0,001874	0,001689	0,001597	0,002275	0,001785	0,002524
prf1	0,001137	0,00093	0,000585	0,00075	0,001106	0,00088	0,000513	0,000631	0,001122	0,001253
mIgM	0,003496	0,006045	0,000874	0,001289	0,002405	0,000777	0,003933	0,003002	0,002762	0,001861
mIgT	0,000538	0,00088	0,000213	0,00075	0,000782	0,000233	0,000681	0,001047	0,000472	0,000216
IgD	0,004275	0,002879	0,000868	0,001061	0,00294	0,000465	0,001797	0,00324	0,001501	0,00147
pax5	0,00147	0,001353	0,00037	0,000431	0,000844	0,000213	0,00162	0,001219	0,000833	0,000601
sIgM	0,042394	0,034915	0,014989	0,004518	0,032577	0,034915	0,028557	0,018073	0,018326	0,048698
sIgT	1,6E-05	2,04E-06	1,29E-06	9,86E-06	5,39E-05	7,22E-06	1,06E-05	9,38E-05	4,72E-05	2,6E-05
nccrp1	0,00194	0,002275	0,002421	0,002472	0,00194	0,002197	0,002064	0,002291	0,002631	0,003496
epx1	0,001586	0,003401	0,00081	0,001822	0,000761	0,001307	0,002244	0,002879	0,003285	0,005336
mpeg1	0,006944	0,007922	0,010167	0,008851	0,00588	0,011598	0,007759	0,00471	0,009552	0,014579

Spleen	RCPT1	RCPT2	RCPT3	RCPT4	RCPT5	RCPT6	RCPT7	RCPT8	RCPT9	RCPT10
zap70	0,00153	0,00142	0,00086	0,001	0,00096	0,00088	0,00136	0,001	0,00088	0,00162
cd3ζ	0,004456	0,004158	0,003377	0,004613	0,003826	0,004334	0,005962	0,00471	0,003308	0,003826
cd4-1	0,000856	0,000644	0,000292	0,000542	0,000194	0,000513	0,00081	0,000434	0,00113	0,000943
cd4-2	0,002631	0,002489	0,00162	0,002577	0,001091	0,001677	0,001797	0,001271	0,002152	0,004645
cd8α	0,001554	0,000911	0,00064	0,001194	0,000649	0,001271	0,001861	0,001362	0,000911	0,000676
cd8β	0,000194	0,000196	8,22E-05	0,000193	7,57E-05	0,000151	0,000485	0,000159	0,000149	0,000106
tbet	0,004216	0,00292	0,002668	0,003799	0,002355	0,003773	0,005563	0,003173	0,004809	0,002507
gata3	0,001887	0,000918	0,000911	0,001785	0,000635	0,001353	0,002079	0,0019	0,001343	0,001565
foxp3	0,000294	0,000499	0,000149	0,000175	0,000144	0,000273	0,000294	0,000169	0,000222	0,000228
tnfα	0,000148	0,000542	0,000111	6,15E-05	0,000141	0,000204	0,000303	0,000443	8,34E-05	0,000147
ifnγ	0,000255	0,000241	0,000241	0,000269	0,000256	0,00029	0,000265	0,00021	0,000177	0,000239
il12 (p40)	2,83E-05	4,05E-05	2,79E-05	1,55E-05	1,5E-05	9,51E-05	0,000109	9,25E-05	5,81E-05	0,000194
il4/13a	6,73E-05	2,1E-05	1,57E-05	4,66E-05	4,59E-05	3,39E-05	0,000106	3,12E-05	6,11E-06	2,64E-05
il4/13b	0,000246	0,000228	0,000316	0,000383	0,00052	0,000277	0,000269	0,000303	0,000143	0,000155
il6	4,54E-06	2,44E-05	1,92E-05	1,84E-06	1,92E-05	1,74E-05	1,47E-05	9,14E-06	2,1E-05	1,74E-05
il10	0,00029	0,00017	0,00017	0,00021	0,0001	0,00015	0,00023	0,00015	0,00013	0,00019
tgfβ	5,13E-05	0,000186	8,28E-05	9,71E-05	0,00013	0,000156	0,00013	0,00015	7,31E-05	0,000255
il17a/f	2,45E-06	4,44E-06	4,23E-06	5,36E-06	2,38E-06	2,08E-05	1,21E-05	1,56E-05	2,66E-05	3,78E-05
gzma	0,000158	6,28E-05	8,81E-05	4,69E-05	0,000111	9,12E-05	9,32E-05	0,00013	4,2E-05	2,95E-05
gzmb	0,001575	0,003151	0,003002	0,0014	0,002228	0,002108	0,002291	0,00324	0,001032	0,001228
prf1	0,00074	0,001202	0,00097	0,000918	0,001061	0,000886	0,001114	0,00088	0,000485	0,000793
mIgM	0,001913	0,001967	0,00282	0,003002	0,002524	0,006003	0,002631	0,00194	0,003799	0,001091
mIgT	0,000216	0,000251	0,000362	0,000236	0,000294	0,000373	0,000513	0,00085	0,000179	0,000109
IgD	0,001169	0,002291	0,002405	0,000899	0,002507	0,00455	0,001334	0,00099	0,001913	0,000465
pax5	0,000456	0,000777	0,00113	0,000667	0,001076	0,001654	0,000772	0,00071	0,000662	0,000365
sIgM	0,041235	0,027776	0,02683	0,048361	0,025916	0,025383	0,057114	0,046714	0,014782	0,02797
sIgT	2,11E-05	7,37E-06	1,56E-05	1,06E-05	5,66E-05	6,32E-05	2,03E-05	3,55E-05	1,7E-05	2,11E-05
nccrp1	0,008851	0,007239	0,004334	0,00471	0,00455	0,002455	0,003217	0,004979	0,002542	0,00286
epx1	0,015843	0,015303	0,009355	0,010598	0,010097	0,003331	0,004809	0,008032	0,003852	0,003545
mpeg1	0,014378	0,01243	0,011281	0,010453	0,00849	0,007705	0,014478	0,006524	0,009355	0,032804

Additional File 2: Table S2. Continued.

Anterior Intestine	CTRL1	CTRL2	CTRL3	CTRL4	CTRL5	CTRL6	CTRL7	CTRL8	CTRL9	CTRL10
zap70	0,00116	0,00107	0,00102	0,00067	0,00095	0,00105	0,001	0,00147	0,00131	0,00098
cd3ζ	0,004129	0,004876	0,00491	0,003594	0,004678	0,004016	0,004776	0,005563	0,00455	0,003852
cd4-1	0,000482	0,000327	0,000273	0,000262	0,000303	0,000788	0,000355	0,000676	0,000577	0,000422
cd4-2	0,000977	0,000672	0,001575	0,000977	0,000502	0,00146	0,001298	0,00149	0,001069	0,001106
cd8α	0,001004	0,000997	0,000844	0,00075	0,001076	0,001178	0,001153	0,001025	0,00142	0,001372
cd8β	0,000139	0,000151	0,000122	8,69E-05	0,000145	0,000189	9,58E-05	0,000103	0,000103	0,00011
tbet	0,000589	0,001061	0,000856	0,000472	0,000725	0,000622	0,000553	0,00075	0,000622	0,000475
gata3	0,001772	0,001161	0,000772	0,001054	0,001391	0,001913	0,001343	0,001236	0,002371	0,001137
foxp3	0,000116	0,00012	0,00013	0,000181	0,00013	0,000255	0,00017	0,000208	0,000161	0,000214
tnfα	4,23E-05	6,63E-05	7,11E-05	5,24E-05	9,45E-05	5,94E-05	0,000164	5,54E-05	9,19E-05	7,11E-05
ifnγ	4,38E-05	5,62E-05	7,41E-05	3,16E-05	4,23E-05	8,11E-05	5,28E-05	5,1E-05	5,06E-05	2,66E-05
il12 (p40)	0,000756	0,000217	0,001532	0,000618	0,000821	0,001153	0,001011	0,001262	0,001061	0,000756
il4/13a	5,9E-05	5,9E-05	4,44E-05	0,000127	0,0002	0,000111	1,45E-05	0,000123	3,12E-05	6,88E-05
il4/13b	0,000816	0,000956	0,000725	0,000793	0,000924	0,000983	0,000745	0,001084	0,000653	0,000812
il6	9,33E-06	1,49E-05	8,29E-06	4,41E-06	1,65E-05	9,52E-06	9,33E-06	3,34E-06	7,07E-06	9,14E-06
il10	0,00013	0,00019	0,0002	0,00015	0,00009	0,00017	0,00027	0,00025	0,00025	0,00015
tgfβ	3,2E-05	1,62E-05	3,12E-05	4,26E-05	3,2E-05	4,41E-05	6,73E-05	4,59E-05	3,71E-05	2,62E-05
il17a/f	4,82E-05	6,69E-06	7,17E-06	3,98E-06	1,34E-06	5,06E-05	2,58E-05	5,5E-05	5,47E-06	2,33E-06
gzma	7,94E-05	5,62E-06	2,58E-05	1,49E-06	8,47E-06	6,41E-05	5,69E-05	9,72E-06	2,89E-05	2,46E-05
gzmb	0,006045	0,001597	0,002291	0,001334	0,003906	0,00296	0,004044	0,002613	0,003331	0,004016
prf1	0,003086	0,001122	0,001677	0,000745	0,002371	0,002291	0,002705	0,001913	0,001211	0,001665
mIgM	0,000217	0,000296	0,000223	0,000177	0,000509	0,000246	0,00032	0,000194	0,000413	0,000265
mIgT	0,000756	0,002524	0,000605	0,000155	0,000827	0,000362	0,000658	0,001575	0,00143	0,00036
IgD	4,89E-05	8,34E-05	0,000163	3,53E-05	0,000233	0,000133	0,000128	6,96E-05	9,92E-05	9,38E-05
pax5	0,00018	0,000523	0,00021	8,81E-05	0,000166	0,000101	0,000126	0,000234	0,000275	7,26E-05
sIgM	0,002613	0,00141	0,000956	0,001522	0,003988	0,002259	0,007189	0,002705	0,002507	0,003619
sIgT	6,45E-05	1,17E-06	2,19E-05	1,39E-05	3,86E-05	3,49E-06	1,16E-05	8,81E-05	0,000135	5,55E-06
nccrp1	0,008315	0,005759	0,007189	0,008373	0,006754	0,007494	0,006615	0,007977	0,009291	0,00568
epx1	0,001797	0,001194	0,001362	0,002371	0,001289	0,003044	0,002687	0,001994	0,00294	0,000874
mpeg1	0,005411	0,003799	0,007759	0,004395	0,00367	0,007652	0,006661	0,009889	0,00588	0,008609

Anterior Intestine	RCPT1	RCPT2	RCPT3	RCPT4	RCPT5	RCPT6	RCPT7	RCPT8	RCPT9	RCPT10
zap70	0,00105	0,00134	0,00215	0,00175	0,00232	0,00226	0,00218	0,00151	0,00133	0,0009
cd3ζ	0,00292	0,003377	0,006087	0,00657	0,007652	0,008669	0,007189	0,010672	0,004645	0,002524
cd4-1	0,000523	0,000527	0,000485	0,000937	0,000667	0,000772	0,000943	0,00088	0,000449	0,000255
cd4-2	0,000943	0,000956	0,000827	0,002244	0,00162	0,00198	0,001586	0,001654	0,001565	0,000983
cd8α	0,000662	0,000715	0,002123	0,002668	0,003086	0,003826	0,00296	0,002705	0,001522	0,000468
cd8β	6,59E-05	8,57E-05	0,00026	0,000341	0,000242	0,00026	0,000345	0,000234	0,000186	8,57E-05
tbet	0,001018	0,001194	0,001609	0,002472	0,00149	0,001575	0,002137	0,001665	0,000874	0,000531
gata3	0,000546	0,001219	0,000937	0,002291	0,000937	0,000766	0,001343	0,001122	0,000658	0,000672
foxp3	0,00013	0,000273	0,000124	0,000286	0,000106	0,000139	0,000239	0,000155	0,000126	0,000105
tnfα	0,000115	0,000123	7,41E-05	5,73E-05	8,87E-05	7,41E-05	0,00015	0,000122	8,45E-05	0,000143
ifnγ	0,000163	0,000307	0,000255	0,000167	7,94E-05	0,000163	0,000233	0,000185	7,11E-05	0,000196
il12 (p40)	0,000362	0,000298	0,000383	0,001032	0,00052	0,000534	0,001084	0,001114	0,000899	0,000182
il4/13a	9,85E-05	5,9E-05	5,9E-05	4,44E-05	0,000127	4,86E-05	0,000138	4,86E-05	0,000129	0,000142
il4/13b	0,000581	0,000816	0,000956	0,000725	0,000793	0,000465	0,001262	0,000597	0,000833	0,000667
il6	2,27E-05	5,06E-05	2,61E-06	2,22E-06	1,55E-06	2,15E-06	1,76E-05	1,21E-05	1,06E-05	5,66E-05
il10	0,00027	0,00025	0,0006	0,00025	0,00056	0,00052	0,00057	0,00035	0,0002	0,00021
tgfβ	3,89E-05	3,18E-05	2,62E-05	2,36E-05	1,79E-05	1,62E-05	5,2E-05	3,09E-05	2,39E-05	2,85E-05
il17a/f	2,44E-05	0,000405	4,11E-05	0,000122	8,51E-05	4,17E-05	3,29E-05	0,000141	3,01E-05	0,000542
gzma	0,000249	0,000265	0,00036	7,67E-05	0,000214	0,000367	0,000593	0,000446	6,5E-05	0,000205
gzmb	0,002388	0,001084	0,00519	0,002182	0,002438	0,003377	0,007813	0,006661	0,002355	0,001848
prf1	0,00144	0,000667	0,003852	0,0019	0,002577	0,004809	0,010167	0,01038	0,002405	0,000804
mIgM	0,000303	0,002339	0,000516	0,00146	0,000443	0,000542	0,000546	0,00019	0,00064	0,001391
mIgT	0,000419	0,000373	0,003002	0,001011	0,001532	0,001926	0,003521	0,001994	0,000557	0,000956
IgD	0,000141	0,00044	0,000397	0,000219	0,000156	0,000168	0,000158	4,82E-05	0,000119	0,000179
pax5	0,000105	0,000549	0,000348	0,000307	0,000275	0,000428	0,000425	0,000256	0,000123	0,000324
sIgM	0,016631	0,15932	0,009099	0,051119	0,020193	0,021793	0,009618	0,005759	0,015303	0,080772
sIgT	4,29E-06	7,96E-07	2,61E-06	1,52E-05	0,000635	0,000234	1,8E-05	1,06E-05	3,34E-06	0,003044
nccrp1	0,015734	0,009753	0,004456	0,005921	0,004072	0,004129	0,006896	0,004809	0,003401	0,004979
epx1	0,000644	0,019237	0,001565	0,010167	0,002438	0,000983	0,001926	0,000605	0,000353	0,009889
mpeg1	0,002093	0,003285	0,003696	0,007652	0,007041	0,00657	0,006754	0,007041	0,006708	0,002108

Additional File 2: Table S2. Continued.

Posterior Intestine	CTRL1	CTRL2	CTRL3	CTRL4	CTRL5	CTRL6	CTRL7	CTRL8	CTRL9	CTRL10
zap70	0,001665	0,001245	0,001018	0,000516	0,000649	0,001307	0,000827	0,003354	0,001835	0,001748
cd3ζ	0,005839	0,004187	0,003065	0,002008	0,003129	0,003424	0,003308	0,003645	0,005263	0,004776
cd4-1	0,001039	0,000686	0,001325	0,000542	0,000233	0,000557	0,000782	0,00099	0,001084	0,000862
cd4-2	0,00198	0,001061	0,001106	0,000605	0,000499	0,001565	0,001186	0,001736	0,00142	0,001362
cd8α	0,001994	0,001039	0,003285	0,001262	0,0014	0,001137	0,001631	0,0041	0,001532	0,001926
cd8β	0,000294	0,000229	0,000394	0,000179	0,000144	0,00018	0,000336	0,000495	0,000244	0,000267
tbet	0,000844	0,000667	0,001353	0,000681	0,001575	0,000918	0,000918	0,000924	0,00097	0,000509
gata3	0,001748	0,001054	0,002339	0,00145	0,000886	0,000649	0,000777	0,001137	0,000856	0,000561
foxp3	0,00032	0,000132	0,000158	0,000152	0,000205	0,000211	0,000265	0,00018	0,000348	0,000452
tnfα	6,96E-05	0,000126	0,000213	7,16E-05	0,000121	6,91E-05	0,000472	0,000194	0,000263	6,5E-05
ifnγ	5,98E-05	2,22E-05	3,53E-05	2,57E-05	5,69E-05	4,05E-05	3,51E-05	3,27E-05	5,03E-05	4,11E-05
il12 (p40)	0,001047	0,000296	0,000631	0,000485	0,000735	0,000735	0,001099	0,001362	0,00097	0,001186
il4/13a	0,000133	9,58E-05	4,5E-05	6,82E-05	0,000159	8,63E-05	6,23E-05	2,38E-05	9,12E-05	5,24E-05
il4/13b	0,000456	0,000388	0,000538	0,000886	0,000856	0,000408	0,00075	0,000513	0,000635	0,000862
il6	5,43E-06	4,54E-06	8,18E-06	2,24E-06	8,18E-06	4,83E-06	6,37E-06	1,3E-05	3,34E-06	1,14E-05
il10	0,000136	0,000133	0,000192	9,12E-05	0,000145	0,000174	0,000456	0,000649	0,000573	0,000217
tgfβ	2,68E-05	6,82E-05	7,21E-05	3,29E-05	4,26E-05	4,63E-05	0,000118	4,29E-05	4,11E-05	2,11E-05
il17a/f	3,48E-05	4,06E-06	2,93E-06	1,15E-05	2,3E-05	4,26E-05	5,43E-05	2,13E-05	3,55E-05	7,78E-05
gzma	2,44E-05	1,6E-05	9,98E-05	3,53E-05	5,39E-05	6,63E-05	4,89E-05	5,13E-05	9,12E-05	3,53E-05
gzmb	0,001887	0,002036	0,005336	0,00147	0,003217	0,003285	0,008201	0,002801	0,003521	0,001848
prf1	0,00194	0,002108	0,002559	0,001522	0,001887	0,001712	0,004395	0,001236	0,002405	0,001039
mIgM	0,001061	0,000327	0,001032	0,000434	0,000804	0,0002	0,000593	0,00145	0,000622	0,000862
mIgT	0,000827	0,001575	0,000557	0,000475	0,002152	0,00071	0,001848	0,001362	0,001011	0,000449
IgD	0,000113	0,000117	0,000205	8,45E-05	0,000431	0,000112	0,000373	0,000569	0,000226	0,000367
pax5	0,000355	0,000157	0,000181	0,000117	0,000462	9,19E-05	0,000223	0,000373	0,00022	0,000416
sIgM	0,014579	0,004843	0,004364	0,012517	0,021493	0,008974	0,027776	0,017701	0,011125	0,011281
sIgT	5,06E-05	8,82E-06	4E-06	5,13E-05	0,000101	1,2E-05	6,37E-06	0,000131	0,000162	6,15E-06
nccrp1	0,005486	0,002355	0,003424	0,00568	0,005119	0,003619	0,00324	0,005336	0,004876	0,003799
epx1	0,006848	0,00198	0,005154	0,001785	0,003645	0,028756	0,008912	0,008315	0,003173	0,010746
mpeg1	0,006087	0,002668	0,004776	0,00265	0,003773	0,005799	0,006087	0,008032	0,007442	0,007705

Posterior Intestine	RCPT1	RCPT2	RCPT3	RCPT4	RCPT5	RCPT6	RCPT7	RCPT8	RCPT9	RCPT10
zap70	0,00142	0,001178	0,001748	0,00128	0,0017	0,003173	0,002123	0,001372	0,001953	0,00143
cd3ζ	0,003545	0,003773	0,003879	0,002705	0,003086	0,00657	0,003262	0,004395	0,003988	0,002244
cd4-1	0,000821	0,000422	0,00145	0,000874	0,00075	0,000725	0,000844	0,001245	0,000905	0,000627
cd4-2	0,001122	0,001511	0,002108	0,001665	0,001153	0,0019	0,001325	0,002108	0,001797	0,000627
cd8α	0,00113	0,000658	0,001362	0,002595	0,001122	0,002595	0,001772	0,00147	0,003401	0,001047
cd8β	0,000201	9,92E-05	0,000265	0,000253	0,00015	0,000241	0,000216	0,000161	0,000378	0,000208
tbet	0,00205	0,000715	0,001575	0,002595	0,001874	0,001724	0,001835	0,000581	0,001501	0,001137
gata3	0,001271	0,000569	0,001381	0,002244	0,001724	0,001307	0,001334	0,000833	0,001362	0,001953
foxp3	0,000161	0,000425	0,000585	0,000201	0,000152	0,000279	0,000269	0,000502	0,000294	0,000124
tnfα	0,000137	0,000152	0,000269	0,00011	0,000174	0,000106	9,25E-05	0,005119	0,000799	0,000327
ifnγ	0,00036	0,000219	0,000577	0,000118	0,000186	0,000132	0,000316	0,000282	7,67E-05	0,000222
il12 (p40)	0,000394	0,000437	0,000181	0,000452	0,000189	0,000614	0,000375	0,001522	0,000977	0,000197
il4/13a	0,000362	0,000452	0,000186	8E-05	0,000434	6,91E-05	0,000816	0,000101	2E-05	0,000112
il4/13b	0,000804	0,000437	0,00071	0,000756	0,000766	0,00044	0,000911	0,000397	0,000527	0,000546
il6	0,000139	1,67E-05	8,4E-05	4,35E-06	2,89E-05	1,48E-05	5,31E-05	0,000267	2,99E-05	6,02E-05
il10	0,000816	0,000286	0,000345	0,000133	0,000446	0,000428	0,00081	0,000408	0,00037	0,000263
tgfβ	3,25E-05	2,62E-05	1,46E-05	3,32E-05	2,5E-05	3,68E-05	1,88E-05	3,2E-05	3,16E-05	2,66E-05
il17a/f	0,000246	0,000411	0,000228	2,71E-05	0,000355	1,65E-05	0,000425	0,000225	9,72E-06	0,000296
gzma	0,001039	0,000631	0,000589	0,000104	0,00093	0,00021	0,00052	0,001228	6,73E-05	0,000416
gzmb	0,002524	0,000899	0,001245	0,001211	0,001343	0,003401	0,001228	0,002152	0,001861	0,001039
prf1	0,001372	0,000597	0,000649	0,001262	0,000816	0,002036	0,000527	0,001091	0,001554	0,001372
mIgM	0,005083	0,002339	0,003401	0,004518	0,002228	0,001353	0,001262	0,002489	0,001372	0,002137
mIgT	0,002275	0,00073	0,001011	0,001145	0,006087	0,000766	0,001501	0,002668	0,000631	0,002388
IgD	0,000672	0,00037	0,001211	0,000309	5,43E-05	0,000405	0,00026	0,001228	0,000316	0,000162
pax5	0,001032	0,000772	0,00093	0,000425	0,000821	0,000388	0,00061	0,00095	0,000296	0,000249
sIgM	0,098073	0,160428	0,178006	0,11744	0,173139	0,073812	0,029977	0,014885	0,025559	0,133046
sIgT	2,93E-06	3,25E-05	3,95E-06	4E-06	0,047696	0,000681	3,56E-06	4,2E-06	2,28E-05	0,007189
nccrp1	0,007922	0,004129	0,004072	0,002559	0,005373	0,002595	0,004216	0,003773	0,003826	0,004245
epx1	0,020333	0,004487	0,021344	0,003285	0,005601	0,002355	0,017098	0,013985	0,002093	0,00324
mpeg1	0,00357	0,005013	0,004809	0,004944	0,004743	0,005799	0,005083	0,00568	0,009618	0,001712
